# Manipulating the Crosstalk between Cancer and Immunosuppressive Cells with Phototherapeutic Gold‐Nanohut for Reprogramming Tumor Microenvironment

**DOI:** 10.1002/advs.202404347

**Published:** 2024-06-23

**Authors:** Hung‐Wei Cheng, Wei Lee, Fei‐Ting Hsu, Yen‐Ho Lai, Shu‐Rou Huang, Chris Seh Hong Lim, Zhen‐Kai Lin, Shih‐Chao Hsu, Chih‐Sheng Chiang, Long‐Bin Jeng, Woei‐Cherng Shyu, San‐Yuan Chen

**Affiliations:** ^1^ Department of Materials Science and Engineering National Yang Ming Chiao Tung University Hsinchu 30010 Taiwan; ^2^ Cell Therapy Center China Medical University Hospital Taichung 40447 Taiwan; ^3^ Department of Biological Science and Technology China Medical University Taichung 406040 Taiwan; ^4^ Translational Medicine Research Center New Drug development Center and Department of Neurology China Medical University Hospital Taichung 40447 Taiwan; ^5^ Department of Physician Assistant Studies School of Health and Rehabilitation Sciences MGH Institute Boston Massachusetts 02114 USA; ^6^ Department of Surgery China Medical University Hospital Taichung 40447 Taiwan; ^7^ Graduate Institute of Biomedical Science China Medical University Taichung 406040 Taiwan; ^8^ Neuroscience and Brain Disease Center China Medical University Taichung 40447 Taiwan; ^9^ Organ Transplantation Center China Medical University Hospital Taichung 40447 Taiwan; ^10^ School of Medicine China Medical University Taichung 406040 Taiwan; ^11^ School of Dentistry College of Dental Medicine Kaohsiung Medical University Kaohsiung 807 Taiwan

**Keywords:** checkpoint immunotherapy, magnetic navigation, photoimmunotherapy, tumor immune microenvironment

## Abstract

Photoimmunotherapy faces challenges due to insufficient intratumoral accumulation of photothermal agents and the reversion of the cancer‐immunity cycle during treatment. In this study, an anti‐PD‐L1‐immobilized magnetic gold nanohut, AuNH‐2‐Ab, with photoresponsive, thermosensitive, and immunomodulatory properties to effectively suppress the growth of primary tumors, elevate immunogenic cell death (ICD) levels, reverse the tumor immune microenvironment (TIME), and consequently inhibit metastases are developed. AuNH‐2‐Ab achieves high tumor accumulation (9.54% injected dose) following systemic administration, allowing the modulation of hyperthermia dose of over 50 °C in the tumor. By optimizing the hyperthermia dose, AuNH‐2‐Ab simultaneously target and eliminate cancer cells and tumor‐associated macrophages, thereby activating potent antitumor immunity without being compromised by immunosuppressive elements. Hyperthermia/pH induced morphological transformation of AuNH‐2‐Ab involving the detachment of the surface antibody for in situ PD‐L1 inhibition, and exposure of the inner fucoidan layer for natural killer (NK) cell activation. This precision photoimmunotherapy approach reprograms the TIME, significantly prolongs survival in a murine hepatocellular carcinoma model (Hep55.1c), and harnesses the synergistic effects of ICD production and checkpoint inhibitors by utilizing a single nanoplatform.

## Graphical abstract

A magnetic photo‐immunotherapy nanoplatform is developed to reprogram tumor immune microenvironment and enhance the response rate along with of efficacy of checkpoint inhibitor by dually targeting to immune suppressing cell and tumor cell.

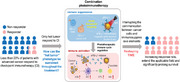

## 1 Introduction

Tumor metastasis, a process that involves the spread of cancer from its primary location to distant sites, remains a major cause of cancer‐related deaths.^[^ ^1^ ^]^ Conventional cancer treatments have shown limited efficacy in inhibiting metastasis.^[^ ^2^ ^]^ Immunotherapy, a treatment that involves the activation of the immune system to kill cancer cells, has emerged as a breakthrough approach in prolonging the survival of patients with advanced cancers. Checkpoint immunotherapy (CI), which restores antitumor immune responses by targeting regulatory pathways, is effective in treating recurrent and metastatic tumors.^[^ ^3^ ^]^ Moreover, exposure of the host immune cells to tumor neoantigens during treatment induces immune memory cells and provides a long‐term immune response to prevent recurrence.^[^ ^4^ ^]^

However, patients with “cold” tumors, where the immune system is minimally activated, have demonstrated low response rate, and hardly benefit from CI treatments.^[^ ^5^ ^]^ In “cold” tumors, tumor‐associated macrophages (TAMs) and regulatory *T*‐cells (Tregs) dominate the niche, creating a suppressive tumor immune microenvironment (TIME) by inhibiting antitumor immune activities. In contrast, the presence of high‐density tumor‐infiltrating lymphocytes (TILs) and the expression of proinflammatory cytokines in a “hot” tumor recruit infiltrative cytotoxic *T*‐cell (CTLs), leading to an enhanced response rate in patients with advanced cancer.^[^ ^6^ ^]^

Increasing immunogenic cancer cell death (ICD)‐derived damage‐associated molecular patterns (DAMPs) has been shown to be a promising therapeutic strategy for converting cold tumors into hot tumors characterized by enhanced activity of antigen‐presenting cells (APCs) and CTLs. Photothermal therapy (PTT) has emerged as an attractive therapeutic approach owing to its ability to induce tumor‐localized DAMPs production in a noninvasive manner with minimal toxicity.^[^ ^7^ ^]^ To facilitate PTT, optical absorbing agents are delivered to the tumor and subsequently irradiated with the corresponding light source such as near‐infrared (NIR) light.^[^ ^8^ ^]^ This process triggers photothermal conversion, which elevates the temperature within or adjacent to the tumor cells, leading to cell death and subsequent release of ICD‐associated signals.

The existing strategy in photoimmunotherapy involves the concurrent administration of CIs or adjuvants with photothermal agents.^[^ ^9^ ^]^ However, the circulation of CI can lead to the blockade of PD‐L1 expression systemically, disrupting the regulatory balance of immunity and causing potential immune‐related adverse effects (irAEs).^[^ ^10^ ^]^ Furthermore, when used in combination with PTT, the dose and regimen of anti‐PD‐L1 vary significantly, making it challenging to establish an optimized combination for translational development. More crucially, the release of DAMPs upregulates PD‐L1 expression on TAMs, which suppresses intratumoral CD8^+^ *T*‐cells, reverts the tumor microenvironment to a relatively cold state, and contributes to tumor progression.^[^ ^11^ ^]^ These weaknesses have impeded the full realization of the benefits from cancer photoimmunotherapy.

In this study, we developed a photoresponsive and thermosensitive magneto‐metal/organic nanostructure to manipulate the crosstalk between cancer and immunosuppressive cells for modulating the TIME and converting it into a ″hot″ status. The nanostructure design comprised a core of gold nanohut (AuNH), a substrate layer consisting of a fucoidan matrix and embedded superparamagnetic iron oxide nanoparticles (IONPs), and an outer layer coated with betanin and immobilized with PD‐L1 antibodies (anti‐PD‐L1). This multi‐layered structure, referred to as AuNH‐2‐Ab, enables the simultaneous targeting and elimination of PD‐L1‐expressing TAMs and tumor cells under NIR irradiation. Unlike existing photoimmunotherapy strategies that primarily focus on promoting anti‐cancer immune cells, AuNH‐2‐Ab can regulate the TIME by inhibiting PD‐L1^+^ TAMs, thereby facilitating the cancer‐immunity cycle to maximize the effect of photoimmunotherapy. In addition, following systemic administration, AuNH‐2‐Ab can be selectively enriched in the tumor region by applying an external magnetic field (eMF) at the tumor site, allowing precise control of the hyperthermia dose within the tumor. This control is crucial for optimizing DAMPs production, which enhances the immune response. Furthermore, the thermal‐ and pH‐sensitive betanin layer can be detached from AuNH‐2‐Ab at elevated temperatures, facilitating the release of anti‐PD‐L1 for penetration into deeper tumor tissue. Subsequently, the exposed fucoidan layer activates natural killer (NK) cells in the TIME, thereby boosting the immune response further.^[^ ^12^ ^]^

AuNH‐2‐Ab confers dual‐targeting capabilities (i.e., anti‐PD‐L1 and magnetic targeting), thereby addressing the challenges associated with the insufficient and non‐specific accumulation of CI.^[^ ^13^ ^]^ Through systematic tailoring of the dual targeting, photo‐responsive, thermosensitive, and immuno‐modulatory properties, AuNH‐2‐Ab effectively suppressed the growth of primary orthotropic hepatocellular carcinoma, elevated ICD levels, reversed TIME by alleviating the PD‐L1‐expressing TAMs, and consequently inhibited metastases. This approach facilitates the nanostructure of AuNH‐2‐Ab to achieve spatiotemporal control over TIME through interactive biological and physicochemical activities, offering a new strategy to effectively modulate the TIME and enhance the therapeutic outcomes of CI in the context of photoimmunotherapy.

## 2 Results & Discussion

### 2.1 Synthesis and Characterization of Magnetic AuNH Nanocomposite Nanostructure (AuNH‐2)

**Scheme 1:**
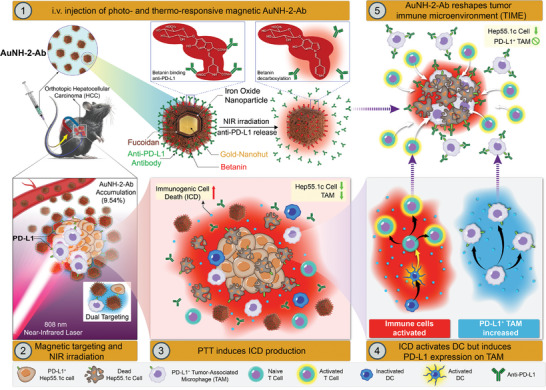
Schematic illustration of the magnetic gold nanohut (AuNH‐2‐Ab) for photothermal immunotherapy. The mechanism of action of AuNH‐2‐Ab demonstrates effective and precise phototherapeutic targeting of cancer cells and tumor‐associated macrophages (TAMs) for modulating tumor immune microenvironment (TIME).

Hybrid iron oxide‐gold nanocomposites have shown potential as theragnostic agents for bimodal cancer cell imaging and PTT;^[^ ^14^ ^]^ however, few studies have addressed the challenges associated with the upregulation of PD‐L1 expression on TAMs when DAMPs are released following treatment. The increased population of PD‐L1^+^ TAMs in the TIME promotes immune escape, thereby reducing the effectiveness of photoimmunotherapy. As depicted in **Scheme** **1**, we developed a photosensitive gold magnetic nanocomposite (AuNH‐2‐Ab) to tackle the obstacle by simultaneous targeting of PD‐L1^+^ cancer cells and TAMs via the PD‐1/PD‐L1 axis. Apart from previous studies, AuNH‐2‐Ab can sustain the immune activation in TIME after PTT by precisely delivering the hyperthermia to both PD‐L1^+^ cancer cells and TAMs and facilitating the controlled release of anti‐PD‐L1 upon heat to attenuate the TAMs. This strategic combination aimed to overcome the challenges associated with increased PD‐L1^+^ TAMs post‐PTT, and was demonstrated using a syngeneic hepatocellular carcinoma (i.e., Hep55.1c) model.

The magnetic AuNH nanocomposite was synthesized via electrostatic self‐assembly, in which anionic fucoidan was coated on a cationic AuNH core and acted as a matrix for embedding cationic IONPs. In contrast to the conventional multistep nucleation and growth processes, our one‐pot strategy simplifies the synthesis and significantly increases the yield of magnetic gold nanohybrids. The resulting fucoidan/IONPs‐coated AuNH (AuNH@F/IO, or AuNH‐1) was further modified to form betanin‐coated AuNH‐1 (AuNH@F/IO@B or AuNH‐2), providing thermoresponsive properties and bioconjugation sites for anti‐PD‐L1. The AuNH nanocomposites (AuNH, AuNH‐1, and AuNH‐2) were colloidally stable when dispersed under aqueous conditions, and their corresponding characteristics are presented in **Table** **1**.

**Table 1:** Characterization of AuNH, AuNH‐1, AuNH‐2, and AuNH‐2‐Ab (*n* = 3).

Sample	Abbreviated name	Diameter [nm]	Zeta potential (Potential [mV])	Polydispersity Index [PDI]
AuNH	AuNH	172.96 ± 8.5	14 ± 2.3	0.189 ± 0.04
AuNH@F/IO	AuNH‐1	195.58 ± 2.6	−29.66 ± 0.44	0.234 ± 0.01
AuNH@F/IO@B	AuNH‐2	213.68 ± 22.9	−19 ± 2.5	0.142 ± 0.08
AuNH@F/IO@B‐anti‐PD‐L1	AuNH‐2‐Ab	255.47 ± 30.17	−21.34 ± 3.2	0.247 ± 0.01

**Figure 1:**
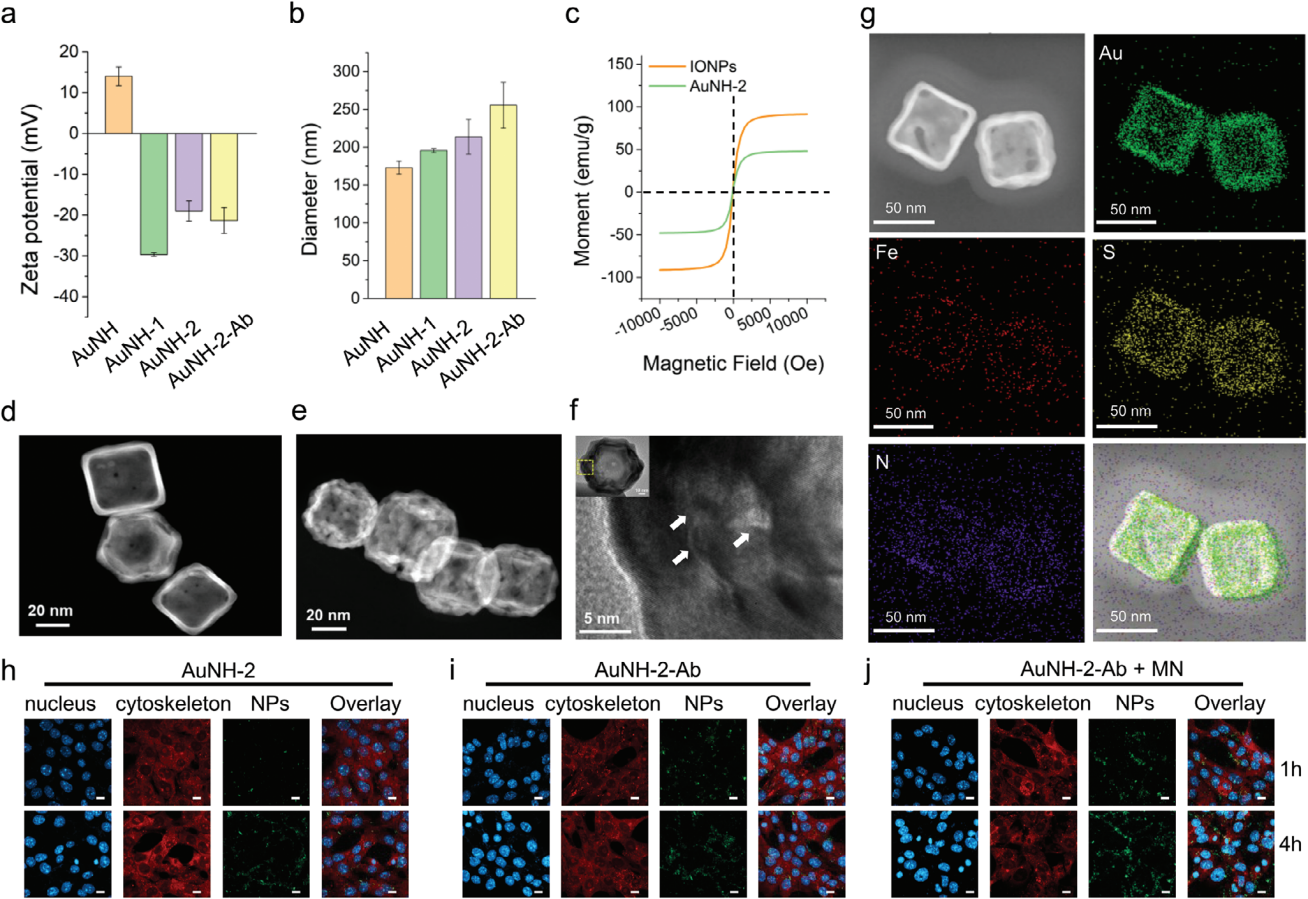
Characterization of the magneto gold/organic nanostructure. a) Zeta potential and b) size of AuNH, AuNH‐1, AuNH‐2, and AuNH‐2‐Ab were measured using dynamic light scattering (DLS). c) Magnetization of IONPs and AuNH‐2. Representative transmission electron microscopic (TEM) images of d) AuNH and e) AuNH‐2. f) IONPs in the shell of AuNH‐2 are indicated with white arrows in the high‐resolution TEM image. g) TEM image and elemental mapping of gold (Au), iron (Fe), sulfur (S), and nitrogen (N) in AuNH‐2‐Ab using TEM and energy‐dispersive X‐ray spectroscopy (EDS). h–j) Cellular uptake of AuNH‐2, AuNH‐2‐Ab, and AuNH‐2‐Ab plus magnetic navigation (MN) at 1 and 4 h incubation visualized using fluorescent microscopy. β‐actin (red) and DAPI (blue) were used to stain the cytoskeleton and nucleus, respectively, and AuNH‐2 was labeled using thiolated FITC‐PEG (FITC‐PEG‐SH, green). *n* = 3 biologically different samples; Scale bar = 10 µm.

The synthesis of AuNH involved the silver nanocube‐displacement method (Figure S1, Supporting Information), followed by surface modification with thiol‐PEG_2000_‐NH_2_ via the gold‐sulfur bond to enable the sequential electrostatic assembly of fucoidan/IONP and betanin. The successful adsorption of each layer was confirmed by changes in the zeta potential (**Figure** **1**a) and size (Figure 1b) of the nanostructures. The AuNHs presented a positively charged surface owing to the presence of a primary amine in PEG.

To form AuNH‐1, we simultaneously added anionic fucoidan and cationic IONPs to the AuNHs. Compared to AuNH with only fucoidan coating, AuNH‐1 presented a more colloid‐stable structure with a higher zeta potential (≈−25 mV) than that of AuNH@F (Figure S2, Supporting Information). The increase in the zeta potential indicated that the IONPs were associated with the coated layer, providing a cationic region for further modification via electrostatic assembly. To enhance the targeting ability, a betanin layer was modified on AuNH‐1 to allow the immobilization of anti‐PD‐L1 through 1‐ethyl‐3‐(3‐dimethylaminopropyl)‐carbodiimide hydrochloride (EDC)/N‐hydroxysulfosuccinimide (NHS) bioconjugation to construct AuNH‐2‐Ab. Bradford assay revealed that ≈40 µg of anti‐PD‐L1 antibody was immobilized on 250 µg AuNH‐2‐Ab.

The incorporation of IONPs into AuNH‐2 conferred it with superparamagnetic properties (Figure 1c), which allowed for the magnetic navigation (MN) of the nanostructures to the tumor site. In contrast to AuNH, AuNH‐2 displayed visible contrast signals from the IONPs on the shell (Figure 1d,e) when analyzed by transmission electron microscopy (TEM). The dark contrast and correlated lattice distance of the IONPs in AuNH‐2 were further observed in the high‐resolution TEM images (indicated by white arrows in Figure 1f), indicating that the IONPs were evenly embedded in the fucoidan matrix. Of note, the AuNH‐2‐Ab demonstrated homogeneous morphology and size as characterized using TEM at a low magnification of 300 kX (Figure S3, Supporting Information).

The elemental distribution of AuNH‐2 was analyzed using energy‐dispersive X‐ray spectroscopy (EDS), confirming the presence of AuNH (Au, labeled as green) mainly dispersed in the core, while IONPs (Fe, labeled as red) was localized in the shell (Figure 1g). Moreover, sulfur (S) and nitrogen (N) signals, which represent the sulfate groups on fucoidan and amines on betanin (C_24_H_26_N_2_O_13_) and anti‐PD‐L1, respectively, were identified to encapsulate the AuNH core, constituting the shell and the outer surface. These findings provide visual evidence of the successful integration of IONPs into AuNH‐2 to achieve MN for enhanced photothermal efficacy.

To assess their photothermal effects, the localized surface plasmon resonance (LSPR) spectra of AuNH and AuNH‐2‐Ab were measured using ultraviolet–visible (UV) spectroscopy (Figure S4, Supporting Information). The LSPR absorption of AuNH‐2‐Ab (≈790 nm) showed a slight shift when compared with that of AuNH, which can be attributed to the surface coating of the materials, including IONPs, fucoidan, and betanin. As the photothermal effect is highly related to colloidal stability, we evaluated the colloidal stability of AuNH‐2 by dispersing it in phosphate‐buffered saline (PBS) and measuring the LSPR spectrum at 0, 1, 4, 8, 12, and 24 h of incubation. The LSPR absorption of AuNH‐2 in PBS consistently peaked at 780–820 nm throughout the 0 to 24 h period. Notably, a ≈5–10% decrease in LSPR absorbance was observed for AuNH‐2 at 24 h compared to 0 h (Figure S5a, Supporting Information). Nevertheless, the photothermal effect remained consistent, following a similar temperature‐time profile, and achieved the same final temperature after 10 min of NIR irradiation (Figure S5b, Supporting Information). These findings confirm that AuNH‐2 maintained high colloidal stability for over 24 h.

We further evaluated the colloidal stability of AuNH‐2‐Ab in 10% FBS‐containing PBS using the same experimental setup. AuNH‐2‐Ab also preserved high colloidal stability from 0 to 24 h, with only a slight decrease in LSPR absorbance from 0.551 at 0 h to 0.491 at 24 h (Figure S5c, Supporting Information). Similarly, upon NIR irradiation, the temperature‐time profile of AuNH‐2‐Ab dispersed in 10% FBS‐containing PBS at 0 and 24 h remained consistent, indicating that the presence of FBS did not compromise the photothermal ability of AuNH‐2‐Ab (Figure S5d, Supporting Information). Importantly, when irradiated with NIR for 5 cycles (in each cycle, AuNH‐2‐Ab was exposed to NIR at 0.5 W cm^−2^ for 10 min followed by a 10 min break at room temperature), the temperature change in each cycle was similar, indicating that the structure and photothermal property remained stable during irradiation (Figure S5e, Supporting Information). These results demonstrate that AuNH‐2‐Ab was colloidally stable in both PBS and 10% FBS‐containing PBS, indicating that the modification with fucoidan and betanin effectively prevents the nanostructure from aggregating.

Facilitating the surface expression of PD‐L1 on Hep55.1c cells (Figure S6, Supporting Information), AuNH‐2‐Ab functionalized with anti‐PD‐L1 demonstrated improved cell association compared to non‐targeted‐AuNH‐2 (Figure 1h,i). Furthermore, when eMF was applied to cells incubated with AuNH‐2‐Ab (AuNH‐2‐Ab plus MN), the fluorescein isothiocyanate (FITC) signal originating from the AuNH‐2‐Ab within Hep55.1c cells was enhanced (Figure 1j; Figure S7, Supporting Information). This indicates that MN facilitated the accumulation of AuNH‐2‐Ab in PD‐L1‐expressing Hep55.1c cells, thereby enhancing its intracellular delivery and potential therapeutic efficacy. The intracellular trafficking studies revealed that after 12 h of incubation, AuNH‐2‐Ab accumulated in both the mitochondria and lysosomes (Figure S8, Supporting Information). Importantly, colocalization with mitochondria facilitates precise delivery of the photothermal effect into cancer cells, allowing for effective apoptosis and ICD for enhanced photoimmunotherapy.^[^ ^15^ ^]^

### 2.2 Photothermic Effect of AuNH‐2‐Ab

In PTT, localized hyperthermia is used to induce cancer cell death at the tumor site. Recent studies have highlighted the crucial role of TIME in determining therapeutic efficacy and prognosis.^[^ ^5^ ^]^ The generation and release of ICD from tumor cells is essential for reversing an immunosuppressive TIME. It has been observed that the expression level of ICD is highly associated with the thermal dose, while a thermal dose that is too high or too low does not result in significant ICD generation, limiting the immunotherapeutic effect.^[^ ^16^ ^]^

To achieve the desired temperature upon NIR irradiation, it relies heavily on the concentration of photothermal agents in the tumor. Previously, intratumoral injection of an adequate amount of a photothermal agent has been employed to modulate the thermal dose.^[^ ^16^ ^]^ However, this approach is not practical in clinical applications for tumors that are located deep within the body. Several studies have shown that successful accumulation of the injected nanoparticles is typically low in the tumor, with a median value of only 0.7% of the injected nanoparticle dose reaching the tumor site.^[^ ^17^ ^]^ Therefore, achieving sufficient intratumoral accumulation of photothermal agents at an optimized thermal dose via intravenous injection remains challenging.^[^ ^18^ ^]^ In contrast, an excessively high temperature in adjacent healthy tissues, resulting from the imprecise accumulation of a photosensitive agent, may induce adverse effects.

**Figure 2:**
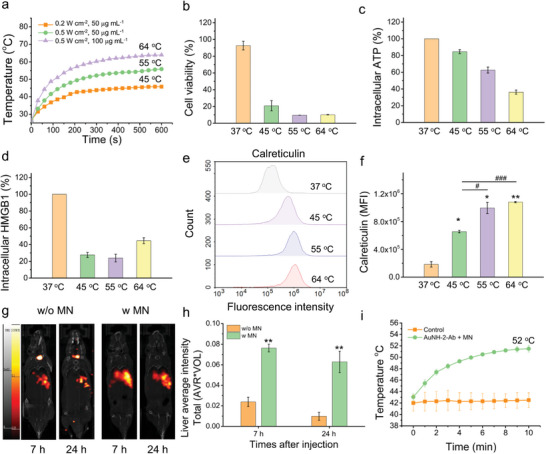
Photothermal conversion efficiency, ICD induction, and biodistribution of AuNH‐2. a) Temperature curve of AuNH‐2 at concentrations of 50 and 100 µg mL^−1^ under NIR (808 nm) irradiation for 10 min at a power density of 0.2 and 0.5 W cm^−2^. b) Viability of Hep55.1c cancer cells treated with AuNH‐2 at different terminal temperatures. The levels of ICD markers including c) adenosine triphosphate (ATP), d) high‐mobility group box 1 (HMGB1), and e,f) calreticulin (CRT) released after phototherapeutic treatment of AuNH‐2. g) Representative nano‐PET/CT images revealing the biodistribution of ^125^I‐labeled AuNH‐2 with and without MN at 7 and 24 h post‐injection. *n* = 2. h) Quantitation of ^125^I‐labeled AuNH‐2 accumulated in liver with/without MN at 7 and 24 h post‐injection. *n* = 2. i) Temperature curves of control and AuNH‐2‐Ab plus MN in liver tissue under NIR irradiation for 10 min at a power density of 0.2 W cm^−2^. All data are expressed as mean ± standard deviation (SD) of at least three biologically different samples, unless otherwise mentioned.

Owing to its magnetic enrichment and tumor‐targeting abilities, AuNH‐2‐Ab overcomes these challenges, and allows for precise accumulation in cancer cells to achieve an adjustable temperature window for optimizing ICD generation. **Figure** **2**a demonstrates the temperature changes in a medium containing AuNH‐2 at different concentrations upon irradiation with an NIR laser (808 nm; power intensity, 0.2 or 0.5 W cm^−2^). The increase in temperature positively correlated with both the concentration of AuNH‐2 and the power intensity of the laser. A temperature plateau was reached around 600 sec post‐irradiation, resulting in terminal temperatures of 64 °C (100 µg mL^−1^, 0.5 W cm^−2^), 55 °C (50 µg mL^−1^, 0.5 W cm^−2^), and 45 °C (50 µg mL^−1^, 0.2 W cm^−2^), respectively, and the variation in plateau temperatures were allowed for subsequent studies on photocytotoxicity and ICD levels.

Both AuNH‐1 and AuNH‐2 exhibited favorable biocompatibility, with cell viability >80% across a concentration range of 15 to 100 µg mL^−1^ in Hep55.1c cells (Figure S9, Supporting Information). In contrast, when subjected to NIR laser irradiation to achieve different terminal temperature ranges, namely ≈64 °C (100 µg mL^−1^, 0.5 W cm^−2^), ≈55 °C (50 µg mL^−1^, 0.5 W cm^−2^), and ≈45 °C (50 µg mL^−1^, 0.2 W cm^−2^), the cells experienced apoptosis and the cell viability decreased significantly (Figure S10, Supporting Information**;** Figure 2b). The levels of ICD‐associated markers, including adenosine triphosphate (ATP) (Figure 2c), high‐mobility group box 1 (HMGB1) (Figure 2d; Figure S11a, Supporting Information), and surface calreticulin (Figure 2e,f; Figure S11b, Supporting Information) were examined in Hep55.1c cells at different terminal temperatures ranges using flow cytometry and microscopy. The results revealed that the cells underwent ICD as the temperature rose from 45 to 64 °C, where a decrease in intracellular ATP and HMGB1 levels accompanying by an increase in surface calreticulin expression was observed. Based on these in vitro results, the optimal thermal window for inducing ICD was determined to be between 55 and 64 °C.

To evaluate the biodistribution of AuNH‐2 and its tumor‐targeting potential, we employed ^125^I labeling and conducted nano‐positron emission tomography‐computed tomography (PET/CT) imaging in luciferase‐expressing Hep55.1c (Hep55.1c‐Luc)‐tumor‐bearing mice. AuNH‐2 accumulated primarily in the liver at 7 h post‐injection, where the tumor was located (Figure 2g) and decreased 24 h post‐injection. MN was performed by applying a magnet to the chest of the mice, a 3.19‐ and 6.39‐fold increase in average ^125^I intensity was observed in the tumor‐bearing livers at 7 and 24 h post‐injection, respectively, compared to that for AuNH‐2 without MN (Figure 2g,h). Further analysis of the biodistribution revealed that AuNH‐2 predominantly accumulated in the liver, spleen, and tumors (Figure S12, Supporting Information). Importantly, the accumulation percentage of AuNH‐2 plus MN reached a high injected dose per gram (%ID g^−1^) of 9.54%, which was significantly higher than that of AuNH‐2 alone (3.04%; *p* = 0.031). Moreover, the tumor‐to‐muscle (T/M) ratio in the AuNH‐2 plus MN group was 2.49‐fold higher than that of the AuNH‐2 group at 24 h (105.2 compared to 42.25), highlighting the enhanced accumulation of AuNH‐2 under MN.^[^ ^19^ ^]^

To investigate the correlation between the photothermal transition efficiency and biodistribution, we conducted NIR irradiation on the liver, where the tumor was located, and monitored the temperature profile. We first measured the temperature of mouse skin treated with PBS under NIR irradiation (0.2 W cm^−2^) and found that the temperature of the skin did not increase significantly. In contrast, when AuNH‐2‐Ab was combined with MN and exposed to NIR irradiation, the skin temperature elevated by 4 °C within 10 min (Figure S13, Supporting Information). As the results indicated that the intensity of NIR irradiation used in the treatment was safe and did not cause skin damage, we performed further experiments using this NIR intensity.

To ensure the success of PTT, it is crucial to achieve appropriate hyperthermia in the tumor tissue while minimizing excessive heating in the adjacent healthy tissues. However, previous studies have highlighted the challenge of achieving sufficient accumulation of photothermal agents in tumors through intravenous administration.^[^ ^18^ ^]^ To address this limitation, the dual‐targeting capability of AuNH‐2‐Ab, involving MN and anti‐PD‐L1, has been employed to improve tumor accumulation and enhance the photothermal effect, while minimizing the risk of thermal damage to adjacent tissues. Hep55.1c‐tumor‐bearing mice were treated with AuNH‐2‐Ab plus MN and irradiated with NIR at a power intensity of 0.2 W cm^−2^ for 10 min at 24 h post‐intravenous injection. The group treated with AuNH‐2‐Ab plus MN exhibited a terminal temperature of 52 °C, specifically localized at the tumor site, as detected using a temperature probe (Figure 2i). This observation suggests that MN effectively facilitated the localization of a higher proportion of nanoparticles to tumors, thereby optimizing the temperature increase. By precisely controlling the temperature range under an eMF, ICD can be effectively induced while minimizing potential damage to the surrounding healthy tissues.

### 2.3 Thermally‐Induced Release of Anti‐PD‐L1 from AuNH‐2‐Ab

**Figure 3:**
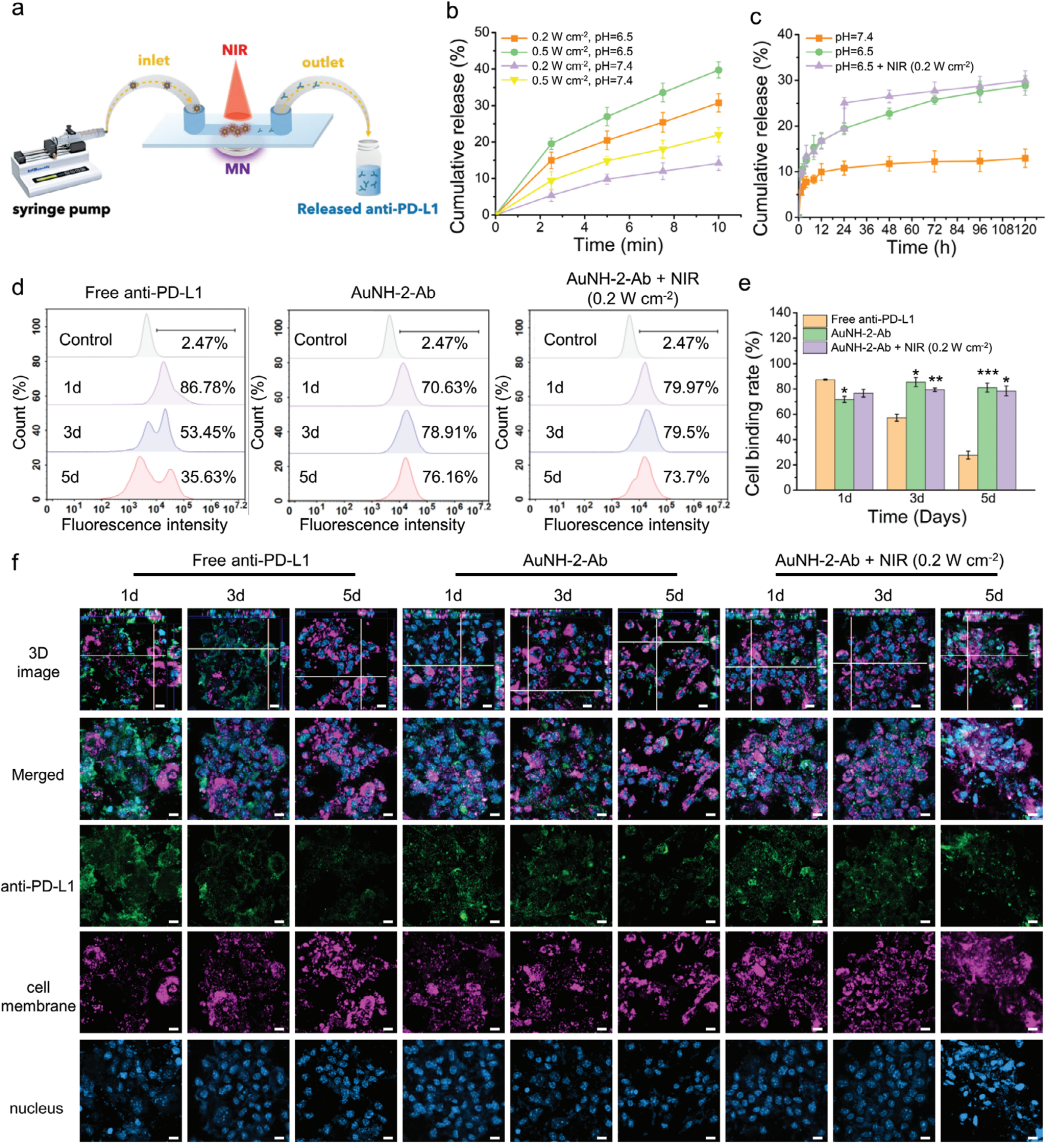
Thermoresponsive release of anti‐PD‐L1 from AuNH‐2‐Ab and its subsequent binding to PD‐L1 on tumor cells. a) A microfluidic channel (MC) system was established to observe the thermoresponsive release of anti‐PD‐L1 from AuNH‐2‐Ab. b) Anti‐PD‐L1 released from AuNH‐2‐Ab incubated in buffers at pH 6.5 or 7.4 and treated with NIR at 0.2 and 0.5 W cm^−2^. Data are expressed as mean ± SD, *n* = 3. c) Thermoresponsive release of anti‐PD‐L1 following treatment with NIR at 24 h post‐incubation in buffer at pH 6.5. d) Flow cytometry was employed to observe the binding rate between Hep55.1c cells and free anti‐PD‐L1, AuNH‐2‐Ab, and AuNH‐2‐Ab plus NIR in a transwell model. NIR was applied for 10 min at 24 h post‐incubation. e) Quantitation of the binding rate was analyzed using flow cytometry. *n* = 3, two‐tailed Student's *t*‐test; ^*^ *p* < 0.05, ^**^ *p* < 0.01, and ^***^ *p* < 0.01. f) The binding rate between Hep55.1c cells and free anti‐PD‐L1, AuNH‐2‐Ab, and AuNH‐2‐Ab plus NIR in a transwell was monitored using fluorescent microscopy. The nuclei were stained with DAPI and anti‐PD‐L1 was labeled with secondary antibody. NIR was applied for 10 min at 24 h post‐incubation. Scale bar = 10 µm.

In addition to the targeting effect of AuNH‐2‐Ab on PD‐L1‐expressing cells, the separation and release of anti‐PD‐L1 from AuNH‐2‐Ab during PTT are critical to achieve effective long‐term checkpoint blockade. To monitor this effect, a microfluidic channel (MC) system consisting of an inlet and an outlet with a magnet positioned underneath the MC was established (**Figure** **3**a). After AuNH‐2‐Ab was injected into the MC through the inlet, NIR of 0.5 W cm^−2^ intensity was applied for 10 min at the location where the eMF was applied. This induced the photothermal effect of AuNH‐2‐Ab that was magnetically enriched in the region, leading to the release of anti‐PD‐L1 from the thermosensitive betanin layer, which was then collected and analyzed using the Bradford assay.

To investigate the effect of pH and NIR intensity on the release of anti‐PD‐L1, AuNH‐2‐Ab was incubated in pH 7.4 and 6.5 buffers under NIR irradiation. The thermosensitive betanin undergoes decarboxylation when exposed to temperatures >50 °C. Under NIR irradiation, the heat generated by the AuNH‐2‐Ab leads to an increase in temperature >50 °C, triggering the detachment of the betanin layer from the nanoparticles, and facilitating the effective release of anti‐PD‐L1 (Figure 3b).

The cumulative release of anti‐PD‐L1 at pH 6.5 environment was measured to be 39.71% and 30.77% under the NIR irradiation at the power of 0.5 and 0.2 W cm^−2^ for 10 min, respectively, indicating that decarboxylation on betanin occurs rapidly upon exposure to hyperthermia. The release of anti‐PD‐L1 at pH 7.4 was slower, indicating that the detachment rate of anti‐PD‐L1 was faster at pH 6.5. This difference in the detachment rate can be attributed to the breaking of the Schiff base bond within the structure, which may be more susceptible to hydrolysis under acidic conditions.

The monitoring of anti‐PD‐L1 release over a period of 120 h revealed a plateau concentration of 12.96% for AuNH‐2‐Ab in pH 7.4 buffer in the dark (Figure 3c). Consistent with the previous results (Figure 3b), an accelerated drug release profile was observed in pH 6.5 buffer in the dark, indicating that the release kinetics are influenced by the pH of the surrounding environment. When AuNH‐2‐Ab was irradiated with NIR (0.2 W cm^−2^) at 24 h, an instant increase in cumulative anti‐PD‐L1 release from 19.5% to 24.8% was detected. This increase in anti‐PD‐L1 release may be attributed to the decarboxylation of betanin during NIR irradiation. These results demonstrate that AuNH‐2‐Ab is capable of rapidly responding to heat and achieving bond cleavage to release anti‐PD‐L1 specifically within the tumor microenvironment upon NIR irradiation.

To investigate the binding efficiency between Hep55.1c cells and freshly prepared anti‐PD‐L1 antibody or anti‐PD‐L1 antibody released from AuNH‐2‐Ab, flow cytometry and confocal laser scanning microscopy (CLSM) was employed. As illustrated in Figure S14 (Supporting Information), a transwell system was used to create two separate chambers: one containing Hep55.1c cells and the other containing either anti‐PD‐L1 antibody or AuNH‐2‐Ab. A membrane was placed between the chambers, allowing the penetration of anti‐PD‐L1 while preventing the passage of AuNH‐2. To facilitate visualization of the antibodies, they were labeled with phycoerythrin for fluorescence detection.

The results showed that freshly‐prepared anti‐PD‐L1 antibody easily penetrated the membrane and bound to Hep55.1c cells, resulting in 86.78% cells conjugated with anti‐PD‐L1 antibodies within 24 h (Figure 3d). However, the fluorescent signal gradually diminished, indicating that the binding gradually decreased over time, while less than 40% of cells remained bound by anti‐PD‐L1 after 5 days of incubation. The anti‐PD‐L1 gradually released from AuNH‐2‐Ab maintained its affinity toward PD‐L1, and 70.63% of Hep55.1c cells in the lower chamber were bound to the released anti‐PD‐L1 after 24 h of incubation. Upon NIR irradiation, the percentage of cells binding further increased to 79.97%, indicating effective controlled release of anti‐PD‐L1 upon thermal stimulus on AuNH‐2‐Ab.

Importantly, the binding rate of freshly‐prepared anti‐PD‐L1 dropped significantly after 3–5 days, but the AuNH‐2‐Ab groups consistently maintained a high cell‐binding rate of over 70% (Figure 3e). Consistent with these findings, CLSM images (Figure 3f) showed a strong fluorescent signal that overlapped with the signal from the cell membrane on Hep55.1c cells in all groups, indicating the binding of anti‐PD‐L1 to PD‐L1 on the cell surface. However, the fluorescent signal diminished over time in the free anti‐PD‐L1 group, while the AuNH‐2‐Ab and AuNH‐2‐Ab plus NIR groups maintained the obvious signal presented on the cell surface. These results further demonstrated that the slow‐release profile from AuNH‐2‐Ab can continuously inhibit PD‐L1 within the tumor microenvironment, preventing the rapid transition from hot tumor to cold tumor as reported in previous literature.^[^ ^20^ ^]^

### 2.4 Therapeutic Efficacy of AuNH‐2‐Ab in Hep55.1c Tumor‐Bearing Mice

To assess the therapeutic efficacy of AuNH‐2‐Ab, Hep55.1c tumor mouse models were divided into groups and treated as follows: anti‐PD‐L1 alone, AuNH‐2, AuNH‐2 plus free anti‐PD‐L1, and AuNH‐2‐Ab with or without MN. All groups were subjected to the same treatment regimen (q4d × 3 schedule). Tumor progression, changes in body weight, survival rates, and lung metastasis were monitored.

**Figure 4:**
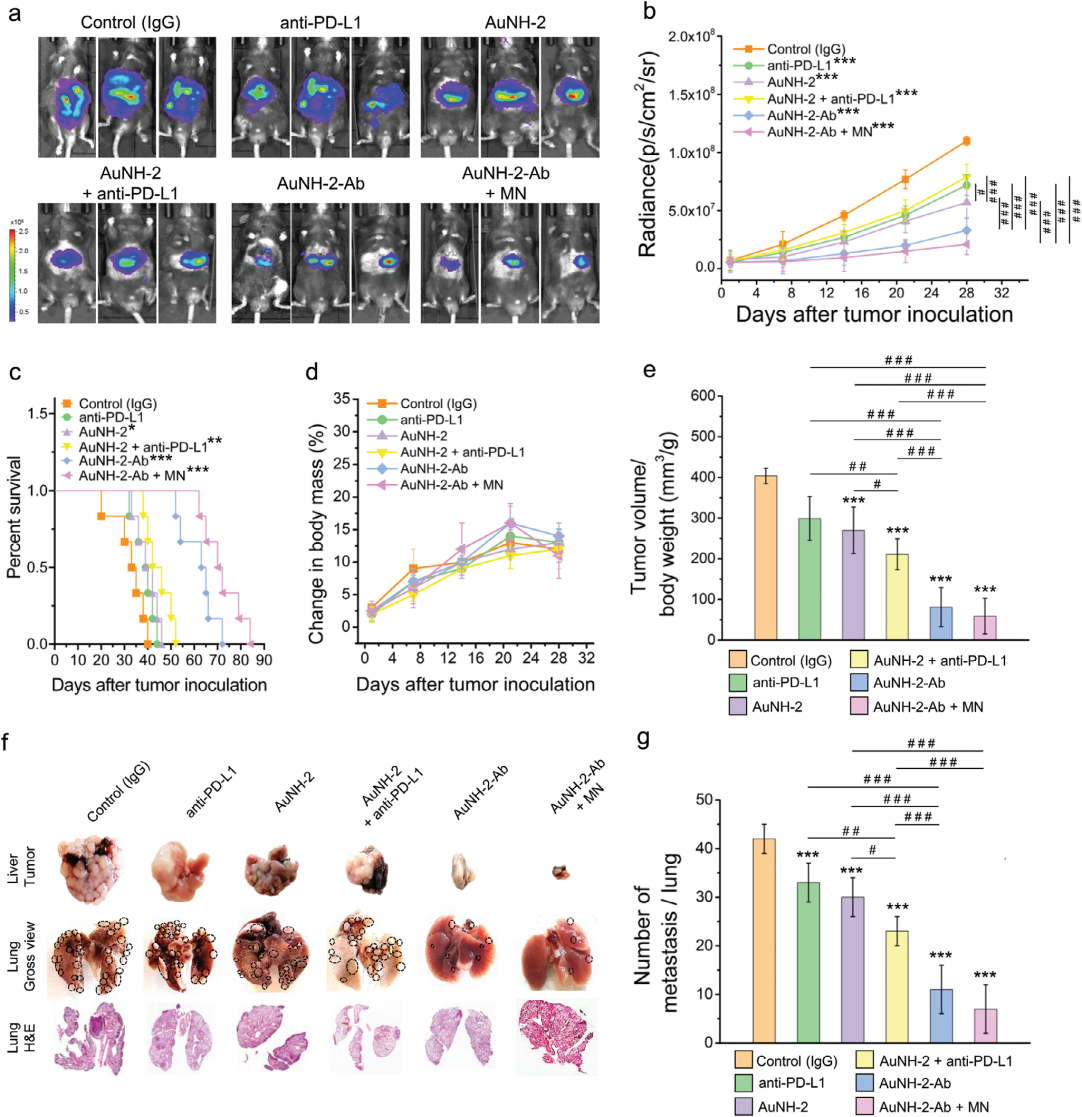
Therapeutic efficacy of various AuNH combinations in inhibiting primary and metastatic Hep55.1c tumor. a) Images of mice from the various treatment groups were obtained using an in vivo imaging system (IVIS). b) Quantitative measurement of the radiance. c) Survival curve of the Hep55.1c‐tumor‐bearing mice treated with control (IgG), anti‐PD‐L1, AuNH‐2, AuNH‐2 plus anti‐PD‐L1, AuNH‐2‐Ab, and AuNH‐2‐Ab plus MN. d) Quantitative analysis of body weight changes, and e) Ratio of tumor volume to body weight in the various treatment groups. one‐way ANOVA with Brown‐Forsythe *post‐hoc* test f) Gross and H&E staining images of the primary and metastatic tumors in the liver and lungs, respectively, from the various treatment groups. Black circles in the gross view of the lungs indicate the metastatic tumor nodules. g) Quantitative analysis of the number of lung metastases. one‐way ANOVA with the Tukey's *post‐hoc* test. All the results are expressed as mean ± standard deviation (SD), *n* = 6; _*_ *p* < 0.05, ^**^ *p* < 0.01, and ^***^ *p* < 0.001 compared with control. ^#^ *p* < 0.05, ^##^ *p* < 0.01, and ^###^ *p* < 0.001 between groups.

The luciferase intensity of the tumors was measured using an in vivo imaging system (IVIS). The semi‐quantitative results demonstrated significant tumor inhibition in the AuNH‐2‐Ab‐treated group compared with that in the groups treated with anti‐PD‐L1, AuNH‐2, and AuNH‐2 plus free anti‐PD‐L1 under NIR irradiation (**Figure** **4**a,b). The median survival of the group treated with the combination of AuNH‐2 and free anti‐PD‐L1, in which the two therapeutic components were administered separately, was ≈44 days. The AuNH‐2‐Ab group showed a significantly prolonged median survival of 64 days (Figure 4c). These results demonstrated that AuNH‐2‐Ab, which simultaneously delivers phototherapeutic agents and checkpoint inhibitors to the tumor, provided improved therapeutic efficacy and prolonged survival. One possible explanation for this is that the AuNH‐2‐Ab formulation concurrently provides both phototherapeutic cytotoxicity and checkpoint inhibition to PD‐L1‐expressing cancers and immunosuppressive cells. Additionally, because nanoparticles larger than 100 nm exhibit limited penetration deep into the tumor tissue,^[^ ^21^ ^]^ the thermoresponsive controlled release of PD‐L1 from AuNH‐2‐Ab compensates for this limitation and enables effective checkpoint inhibition throughout the tumor.

MN application at the tumor site further significantly suppressed tumor progression, extending the median survival by over 70 days in the AuNH‐2‐Ab plus MN group. Importantly, the body weight of the treated animals was not significantly different from that of the animals in the control group, indicating all the treatments demonstrated an acceptable safety profile (Figure 4d). The tumors collected from the mice treated with AuNH‐2‐Ab and AuNH‐2‐Ab plus MN showed a smaller tumor volume‐body weight ratio (Figure 4e), which was consistent with the results shown in Figure 4b,c.

At day 28 post‐tumor inoculation, the mice were sacrificed, and the tumors were collected for histological analysis (Figure S15a, Supporting Information). To verify apoptotic cell death in the tumor, we performed TUNEL staining of the tumor tissues. As shown in Figure S15b (Supporting Information), the treatment of AuNH‐2‐Ab or AuNH‐2‐Ab plus MN can induce regional cell apoptosis under NIR irradiation. More importantly, the combination of AuNH‐2‐Ab plus MN with NIR irradiation exhibited more dispersed TUNEL signals across the tissues, indicating that the dual targeting delivery of AuNH‐2‐Ab plus MN (i.e., PD‐L1 targeting and magnetic targeting) to the tumor tissue can enhance the efficacy of photothermal therapy. More importantly, at the meantime, a significant reduction in lung metastasis was observed in mice treated with AuNH‐2‐Ab plus MN, which had fewer than 5 nodules compared to 42, 33, 30, 23, and 11 nodules in the control, anti‐PD‐L1, AuNH‐2, AuNH‐2 plus anti‐PD‐L1, and AuNH‐2‐Ab groups, respectively (Figure 4f,g). These results suggest that the combination of AuNH‐2‐Ab and MN not only suppresses tumor growth but also inhibits the spread of cancer cells to the lungs.

### 2.5 Reversing Suppressive TIME with AuNH‐2‐Ab‐Driven Photoimmunotherapy

The gating strategies for Tregs, TAMs, subsets of T cells, DCs, and NK cells in the tumors of mice treated with IgG (control) and AuNH‐2‐Ab plus MN were presented in Figures S16, S17, and S18 (Supporting Information), respectively. Treatment with AuNH‐2‐Ab substantially restored the percentage of tumor‐infiltrating CD45^+^ leukocytes compared with that in the control, anti‐PD‐L1 alone, AuNH‐2, and AuNH‐2 plus anti‐PD‐L1 groups (Figure S19, Supporting Information). This suggests that AuNH‐2‐Ab treatment positively affects the immune cell composition within the tumor, potentially enhancing the immune response against the tumor.

Although PTT can activate the immune system, its effects are only sustained in a limited period.^[^ ^6^, ^22^ ^]^ Hyperthermia‐induced immune response has been reported to be attenuated by TAMs present in the TIME, thereby limiting the efficacy of photoimmunotherapy. To address this challenge, current clinical practice involves a combination of PTT and concurrent CIs.^[^ ^23^ ^]^ Furthermore, the analysis revealed that within the Hep55.1 hepatoma tumor, TAMs expressing PD‐L1 accounted for ≈39.5% of the total TAM population (Figure S20, Supporting Information).^[^ ^24^ ^]^ The presence of PD‐L1‐expressing TAMs contributes to an immunosuppressive environment in tumors.^[^ ^25^ ^]^ To investigate the potential of thermally‐induced release of anti‐PD‐L1 from AuNH‐2‐Ab in enhancing antitumor immunity and overcoming these obstacles, we examined the dynamics of DCs, CTLs, helper T cells, memory T cells, Tregs, and TAMs following various treatments using a q4dx3 course at four weeks after tumor inoculation.

**Figure 5:**
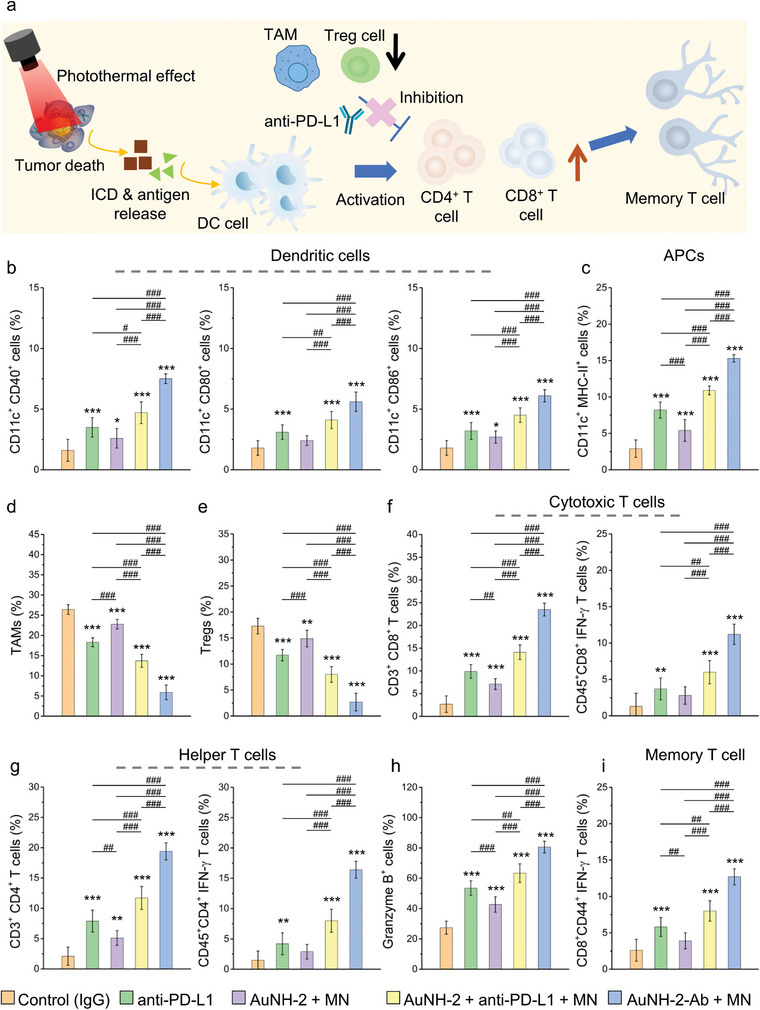
Photoimmunotherapy with AuNH‐Ab treatment. a) Schematic illustration of the immune response during AuNH‐2‐Ab treatment. Quantitative assessment of immune cells including b) DC (CD11c^+^CD40^+^ CD11c^+^CD80^+^ and CD11c^+^CD86^+^), c) APC (CD11c^+^MHC‐II^+^), d) TAMs, e) Tregs, f) T cytotoxic cells (CD3^+^CD8^+^, CD45^+^CD8^+^ IFN‐γ), g) T helper cells (CD3^+^CD4^+^, CD45^+^CD4^+^IFN‐γ), h) Granzyme B^+^ positive cell, and i) memory T cells (CD8^+^CD44^+^IFN‐γ) in the TIME after various treatments at four weeks after tumor inoculation. All data were presented in mean ± SD. One‐way ANOVA with the Tukey's *post‐hoc* test; *n* = 6. ^*^ *p* < 0.05, ^**^ *p* < 0.01, and ^***^ *p* < 0.001 compared with control group and ^#^ *p* < 0.05, ^##^ *p* < 0.01 and ^###^ *p* < 0.001 between groups.

As shown in **Figure** **5**a, PTT induces hyperthermia in cancer cells, leading to the release of danger signals. DCs captured the danger signals and initiated downstream immune responses. However, the presence of PD‐L1 in tumors inhibits antitumor immune cell populations. Treatment with anti‐PD‐L1 alone leads to the suppression of Tregs and TAMs, consistent with the known reprogramming effects of anti‐PD‐L1 in the TIME. Despite this effect, the activation of DCs and cytotoxic *T*‐cells remain limited owing to restricted exposure to both ICD and tumor antigens (Figure 5b–e). Similarly, in the absence of the PD‐L1 blockade in the TIME, treatment with AuNH‐2 results in only a modest enhancement of DCs and T cells.

In contrast, the combination of MN and AuNH‐2‐Ab substantially increased the accumulation of nanoparticles at the tumor site. This synergistic approach facilitated multiple mechanisms of action that modulate tumor immunity. First, AuNH‐2‐Ab targeted both PD‐L1‐expressing Hep55.1c cells and pro‐tumor immune cells simultaneously, and the application of MN in conjunction with PTT induced sufficient hyperthermia, leading to the elimination of these cells within the tumor. Additionally, the thermoresponsive controlled release of anti‐PD‐L1 in the TIME can effectively block intratumoral PD‐L1 expression in the long‐term, thereby maintaining a “hot” tumor status within the TIME. By combining these strategies, PD‐L1 checkpoints can be effectively blocked, resulting in the initiation of a robust and long‐lasting immune response.

Our results revealed a substantial increase in mature DCs expressing CD11c^+^CD40^+^, CD11c^+^CD80^+^, and CD11c^+^CD86^+^ markers as well as in APCs expressing CD11c^+^MHC‐II^+^ with fold changes of 4.7, 3.1, 3.4, and 5.2, respectively, compared with that in the control group (Figure 5b,c). Meanwhile, the treatments also resulted in a significant attenuation of TAMs and Tregs (Figure 5d,e), primarily caused by the local release of anti‐PD‐L1 from AuNH‐2‐Ab. Additionally, we observed a systemic reduction in Tregs in the spleen compared with single and other combination therapies (Figure S21, Supporting Information).

The population of effective *T*‐cells, including CD3^+^CD8^+^ and CD45^+^CD8^+^IFN‐γ^+^ TILs showed a significant increase with fold changes of 8.7 and 8.6, respectively, compared to that in the control group (Figure 5f). Similarly, helper *T*‐cells, represented by CD3^+^CD4^+^ and CD45^+^CD4^+^IFN‐γ^+^ TILs (Figure 5g), exhibited a robust expansion in the primary tumors, with fold changes of 9.2 and 16.4, respectively, compared to that in the control group. Notably, the substantial increase in intracellular granzyme B (Grb^+^) positive cell indicated that the *T*‐cells induced by the treatment possessed cytotoxic functions (Figure 5h). While the combination of anti‐PD‐L1 and AuNH‐2 also stimulated the activation of DCs and antitumor *T*‐cells, its efficacy was significantly lower than that of the delivery of the two components using the integrated AuNH‐2‐Ab system. Furthermore, we identified the activation of NK cells (CD3^−^NK1.1^+^, KLRG1^+^NK1.1^+^) in the primary tumors (Figure S22, Supporting Information), demonstrating the engagement of innate immune antitumor responses following the treatment with AuNH‐2‐Ab compared to that in the other treatment groups. These results suggest that the presence of fucoidan in AuNH‐2‐Ab effectively activates NK cells. Importantly, a significant increase in tumor‐specific CD8^+^CD44^+^IFN‐γ^+^ *T*‐cells with central memory potential was observed (Figure 5i),^[^ ^26^ ^]^ which can be attributed to the tumor enrichment effect by MN application.

The ratio of the anti‐ and pro‐tumor immune populations was calculated and shown in Figure S23 (Supporting Information). The results demonstrated a high ratio of CD8^+^ and CD4^+^ *T*‐cells to Tregs and TAMs, which is associated with a cell‐mediated antitumor effect and improved prognosis, respectively. Treatment with AuNH‐2‐Ab plus MN not only enhanced antitumor immunity by initiating the cancer‐immunity cycle and amplifying the *T*‐cell immune response, but also reversed the immunosuppressive TIME.

### 2.6 Reduction of the Immune‐Related Adverse Events (irAEs)

**Figure 6:**
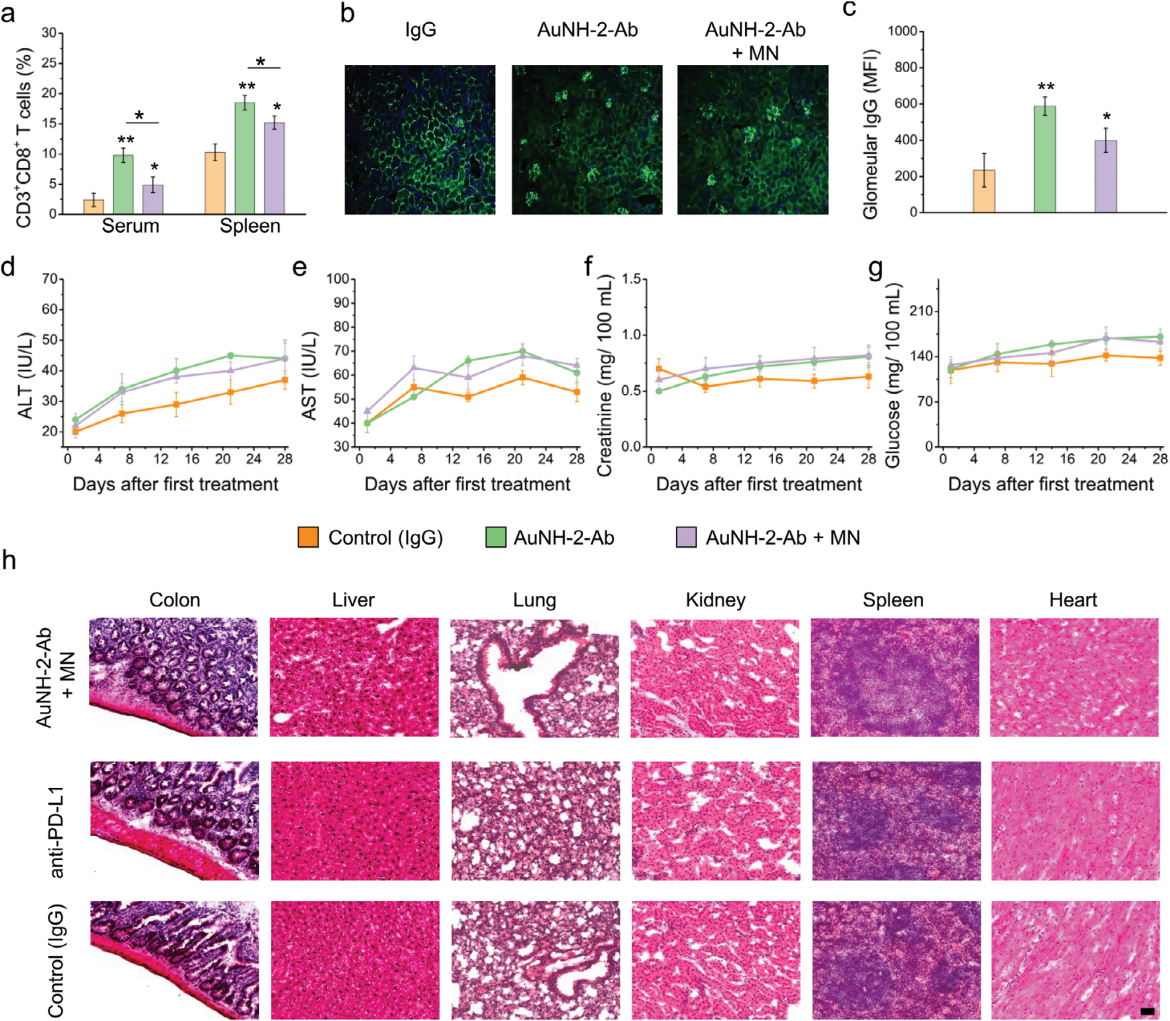
Assessment of the safety profile in mice following various treatments. a) Quantitative analysis of immunological profiles including CD3^+^CD8^+^ T cells in serum, and spleen, respectively, following treatment with AuNH‐2‐Ab with or without MN. *n* = 6. b) Representative images, and c) quantitative comparison of immunohistochemical analysis of IgG deposition in glomeruli in AuNH‐2‐Ab treatment with or without MN. *n* = 6. Quantitative measurement of biochemical parameters including d) ALT, (e) AST, f) creatinine, and g) glucose values by serum analysis in mice over time after treatment with IgG (control), AuNH‐2‐Ab, and AuNH‐2‐Ab plus MN. *n* = 6. h) Histological assessment of major organs using hematoxylin and eosin (H&E) staining following treatment with control (IgG), anti‐PD‐L1 plus MN, and AuNH‐2‐Ab plus MN. (Scale bar = 50 µm). One‐way ANOVA with the Tukey's *post‐hoc* test; ^*^ *p* < 0.05 and ^**^ *p* < 0.01 between groups.

The systemic toxicology and immune‐safety of AuNH‐2‐Ab were thoroughly evaluated, including body weight monitoring, renal IgG deposition, biochemical functions, and histological analysis. The application of MN along with AuNH‐2‐Ab effectively reduced systemic CD3^+^CD8^+^ *T*‐cells in both the serum and spleen, resulting in 2‐ and 1.16‐fold, respectively, compared with AuNH‐2‐Ab without MN (**Figure** **6**a). Additionally, we observed a higher level of glomerular IgG deposition after treatment with AuNH‐2‐Ab alone (Figure 6b,c). However, when MN was applied, the amount of deposition was reduced, indicating that the use of MN can minimize the random distribution of anti‐PD‐L1, thereby increasing the safety profile of the CIs.

Furthermore, hepatic enzymes (AST/ALT), kidney function index (creatinine), and glucose levels were within the reference values following various treatments (Figure 6d–g). Histological analysis of the major organs, including the colon, liver, lung, kidney, spleen, and heart revealed minor‐to‐no lesions following treatment (Figure 6h; Figure S24, Supporting Information). Notably, NIR irradiation did not induce any damage in the major organs, particularly the liver, indicating that a favorable safety window can be achieved by the selectivity of AuNH‐2‐Ab and the precise localization of NIR irradiation at the tumor site. These findings highlight the effective alleviation of irAEs with the application of MN and low‐intensity NIR radiation, thus minimizing the risk of damage to adjacent and systemic tissues.

## 3 Conclusion

In summary, our study demonstrated the successful development of a novel photothermal immunotherapy modality using AuNH‐2‐Ab immobilized with an immune checkpoint inhibitor. This innovative approach effectively converts a “cold” tumor into a “hot” tumor by addressing the limitations of insufficient nanoparticle accumulation and limited immune activation. The use of MN enabled optimization of the terminal temperature and thermal dose during PTT, maximizing the production of ICD markers in the tumor. Furthermore, AuNH‐2‐Ab played a dual role in attenuating TAMs by directly targeting them with PTT and blocking the immune checkpoint PD‐L1 through the heat‐induced release of anti‐PD‐L1. This strategy complemented the cancer‐immunity cycle and reprogramed the TIME in hepatoma. By rebalancing the antitumor and pro‐tumor immune populations in the TIME and activating memory *T*‐cells, our approach demonstrated the ability to eliminate local and metastatic tumors, leading to significantly improved therapeutic outcomes. The approach with the simultaneous precision targeting of tumors and pro‐tumor immune cells using AuNH‐2‐Ab represents a promising avenue for precise photoimmunotherapy.

## 4 Experimental Section

### Preparation of Gold Nanocages (AuNCs) and AuNHs

AuNHs were synthesized based on the method described by Xia et al.^[^ ^27^ ^]^ Initially, ethylene glycol (EG, 6 mL) was placed in clean reaction vials and preheated in an oil bath at 150 °C for 1 h. Next, two different amounts of Na_2_S (3 mm, 70 µL or 100 µL) were slowly added to the EG in the vials over 8 min. Then, 1.5 mL of poly vinyl pyrrolidone (PVP, 0.18 m) and AgNO_3_ in EG (0.28 M) were mixed, and allowed to react for 15 min. The mixture was allowed to cool to room temperature, and the silver nanocubes were precipitated by adding acetone and collected by sequential centrifugation at 2000 ×g for 30 min and 9000 ×g for 10 min. The resulting Ag nanocubes were dispersed in deionized water (DI, 4 mL) and stored at 4 °C.

For the synthesis of AuNHs, silver nanocubes (2 mL) and PVP (9 mm, 10 mL) solutions were added to a double‐neck flask. Then, HAuCl_4_ solution (0.2 mm) was added to the mixture at a rate of 10 mL h^−1^ until the desired localized surface plasmon resonance (LSPR) peak of AuNH was achieved. Color changes were observed and progress of the synthesis was monitored with a UV–VisUV–visible spectrophotometer (Evolution 300, Thermo Fisher Scientific). After completion of the reaction, excess NaCl crystals were added to the mixture, followed by three washes and centrifugation (11,000 rpm for 10 min). The resulting AuNCs were dispersed in DI water (2 mL) and stored at 4 °C.

### Multilayer Assembly of AuNH‐2

AuNHs were synthesized using a multilayer assembly method. First, AuNH‐NH_2_ was synthesized by forming a gold–sulfur bond. AuNH (1 mg mL^−1^) was mixed with thiol‐PEG2000‐NH_2_ (6 mg mL^−1^) and shaken (200 rpm) at 4 °C overnight. The resulting AuNH‐NH_2_ were collected by centrifugation at 11 000 rpm for 10 min, dispersed in DI water (250 µg mL^−1^), and stored at 4 °C. To visualize and quantify the anti‐PD‐L1, thiolated FITC‐PEG (FITC‐PEG‐SH) was mixed with thiol‐PEG2000‐NH_2_ to immobilize FITC‐PEG‐SH onto AuNHs through a gold‐sulfur bond.

IONPs were synthesized following the method of Mikhaylova et al.^[^ ^28^ ^]^ For multilayer assembly, IONPs (1 mg mL^−1^) were mixed with AuNH‐NH_2_ and shaken (200 rpm), and then fucoidan (50 mL, 1 mg mL^−1^, Sigma) was added at a rate of 20 mL h^−1^ into the mixture, followed by centrifugation with DI water to remove excess polyelectrolyte to form AuNH‐1. The resulting product was purified using a magnetic selection equipment (MagniSort, ThermoFisher, USA) to remove excess polyelectrolyte and then resuspended in distilled DI water or 0.1 m buffer (pH, 6) for further surface modification. For surface modification, betanin (10 mg mL^−1^, Sigma) in a 0.1 m buffer solution (pH = 6) was added to AuNH‐1 (Au concentration at 250 µg mL^−1^) and stirred at 4 °C overnight. Following completion of the reaction, the products were washed three times and resuspended in 0.1 m buffer (pH = 6) using the magnetic selection equipment.

### Synthesis of AuNH‐2‐Ab

To synthesize AuNH‐2‐Ab, anti‐PD‐L1 was immobilized on the surface of AuNH‐2 using the 1‐ethyl‐3‐(3‐dimethylaminopropyl)‐carbodiimide hydrochloride (EDC) and N‐hydroxysulfosuccinimide (NHS) bioconjugation method. Initially, EDC (0.5 mL, 200 mm, Sigma) was mixed with AuNH‐2 (1 mL, Au concentration at 250 µg mL^−1^) in an 8 mL glass vial, which was placed in a styrofoam box with ice and fixed on an orbital shaker (OS701, BioPioneer Tech CO., Ltd, Taiwan) set at 200 rpm for 2 h. Subsequently, NHS (0.5 mL, 200 mm, Sigma) and anti‐PD‐L1 (50 µg, Bio X Cell) were added to the mixture and incubated at 4 °C for 30 min. The resulting AuNH‐2‐Ab was purified magnetically using a magnetic selection apparatus (MagniSort, ThermoFisher, USA) to remove any unconjugated anti‐PD‐L1. Finally, the purified product was resuspended in a buffer (pH 6, 0.1 m) and stored at 4 °C for further use.

### Characterization of AuNH‐2‐Ab

The morphology of AuNH‐2 was observed using scanning electron microscopy (SEM) and transmission electron microscopy (TEM). The nanoparticle size was analyzed by dynamic light scattering (DLS) using a Delsa Nano C (Beckman Coulter Inc.) particle analyzer. The variation in zeta potential of these nanocapsules was measured using the same particle analyzer (Delsa Nano C; Beckman Coulter Inc.). The optical properties of AuNH‐2 were measured using ultraviolet–visible (UV–Vis) spectroscopy (Epoch 2 Gen5, BioTek).

### Bradford Assay

To measure the concentration of the immobilized antibodies, Bradford reagent (40 µL) was mixed with various concentrations of the BSA standard (2.5, 5, 10, 12.5, 20, and 25 µg mL^−1^), and the absorbance was measured to generate a calibration curve. After immobilizing the antibodies on AuNH‐2‐Ab and removing the unconjugated antibodies using magnetic selection equipment, the protein concentrations in the samples were measured at 595 nm using an ELISA reader (Epoch 2 Gen5, BioTek). The concentrations of the target antibodies were calculated based on the calibration curve.

### Anti‐PD‐L1 Release from AuNH‐2‐Ab at Different pH and Temperature

AuNH‐2‐Ab was suspended in PBS solutions at pH 6.5 and 7.4, and subjected to NIR irradiation at two different power intensities (0.2 and 0.5 W cm^−2^) for 10 min using a µ‐Slide I 0.8 Luer system with PBS flow rate of 12 mL h^−1^. Samples were collected at specific time points (2.5, 5, 7.5, and 10 min) during the NIR irradiation.

For long‐term release study, AuNH‐2‐Ab was placed in PBS solutions at different pH values (pH 6.5 and 7.4) at 37 °C and 100 rpm for various time intervals (1, 2, 4, 8, 12, 24, 48, 72, 96, and 120 h). The samples were collected at each time point using magnetic selection equipment (MagniSort, eBioscience) and re‐dispersed in DI water. The concentration of anti‐PD‐L1 was determined using Bradford assay (Thermo X Series II).

Additionally, NIR irradiation (0.2 W cm^−2^) (LSR‐PS‐FA, Anjun) was applied to AuNH‐2‐Ab samples at 24 h to simulate the treatment in vivo. Samples were collected at various time points (1, 2, 4, 8, 12, 24, 48, 72, 96, and 120 h) after the NIR irradiation.

### Cell Culture

The Hep55.1c (RRID: CVCL_5766) mouse liver cancer cell line was provided by China Medical University in Taiwan. The cells were maintained in Dulbecco's modified Eagle's medium (DMEM) supplemented with 10% fetal bovine serum (FBS) and 1% penicillin‐streptomycin.

### Cellular Uptake of AuNH‐2‐Ab

Hep55.1c cells (5 × 10^4^ in pre‐well) were seeded on microscope slide coverslips in 24‐well plates and allowed to adhere for 24 h. Subsequently, the cells were incubated with labeled AuNH‐2 and AuNH‐2‐Ab for 1h and 4 h. In vitro magnetic navigation (MN) was conducted with cells incubated with the labeled AuNH‐2 for 30 min, and samples were collected at 1 and 4 h. After incubation, the nucleus was stained with DAPI, and the cytoskeleton was stained with β‐actin. The subcellular localization of AuNH‐2 and AuNH‐2‐Ab was visualized using a CLSM (Carl Zeiss LSM 510, Carl Zeiss, Jena, Germany) and quantified using a Novocyte Flow Cytometer (ACEA Biosciences). In addition, to observe the cell trafficking of AuNH‐2‐Ab, the cells were incubated with AuNH‐2‐Ab and collected at 12 h. After incubation, MitoTracker Red CMXRos (Thermo Fisher Scientific, USA) and Recombinant Anti‐LAMP2 antibody (ab125068, Abcam). The cells were then incubated with the corresponding (Goat Anti‐Rabbit IgG H&L (Alexa Fluor 594, A‐11012) to bind the lysotracker for detection, and the nuclei were stained with DAPI for 15 min. Samples were visualized using a CLSM (Carl Zeiss LSM 510, Carl Zeiss, Jena, Germany)

### Cytotoxicity Assay in Hep55.1c Cells Treated with PTT

Hep55.1c cells (1 × 10^4^ in pre well) were cultured in 96‐well plates for 24 h prior to cytotoxicity testing. Two different concentrations of AuNH‐2 (50 and 100 µg mL^−1^ in FBS‐free medium) were added to the wells and incubated for 24 h. After incubation, the Hep55.1c cells were treated with an NIR laser (808 nm) at two intensities (0.2 and 0.5 W cm^−2^) for 10 min, while the control group received no treatment. Following treatment, the cells were further incubated for 1 day. The Cell Counting Kit‐8 (CCK‐8) reagent (10%) was then added to each well and incubated for 1 h and the absorbance of the wells was measured at 450 nm using an ELISA reader (Epoch 2 Gen5, BioTek) to quantify cell viability.

In addition, to evaluate the cytotoxicity of AuNH‐1 and AuNH‐2, the cells were incubated with an FBS‐free medium containing various concentrations of AuNHs (15, 30, and 50, 100 µg mL^−1^) for 24 h. After incubation, the CCK‐8 reagent (10%) was added to each well and incubated for 1 h, and the absorbance was measured at 450 nm using the ELISA reader (Epoch 2 Gen5, BioTek) to assess cell viability.

### Apoptosis Assays

Hep55.1c cells (1 × 10^4^ in pre‐well) were cultured in 96‐well plates for 24 h prior to apoptosis assays. Two different concentrations of AuNH‐2 (50 and 100 µg mL^−1^ in FBS‐free medium) were added to the wells and incubated for 24 h. After incubation, the Hep55.1c cells were treated with an NIR laser (808 nm) at two intensities (0.2 and 0.5 W cm^−2^) for 10 min, while the control group received no treatment. Following treatment, the cells were further incubated for one day. The cells were harvested by trypsin‐EDTA and stained according to protocol of BD Pharmingen FITC Annexin V Apoptosis Detection Kit I (RRID:AB_2 869 265. Samples were analyzed on a Flow Cytometer (Attune NxT, Thermo Fisher Scientific, USA).

### Expression of Immunogenic Cell Death (ICD) Markers in Hep55.1c Cells Treated with PTT

Hep55.1c cells (2 × 10^5^ in pre well) were cultured in 96‐well plates for 24 h before testing for ICD markers. Two different concentrations of AuNH‐2 (50 and 100 µg mL^−1^ in FBS‐free medium) were added to the wells and incubated for 24 h. After incubation, the cells were treated with NIR (808 nm) at two power intensities (0.2 and 0.5 W cm^−2^) for 10 min, while the control group received no treatment. Following the treatment, the cells were incubated for 4 h, washed, stained with Alexa Fluor 488 conjugated anti‐calreticulin antibody (ab196158, Abcam), and subjected to flow cytometric (Novocyte Flow Cytometer, ACEA Biosciences, USA) analyses. To analyze intracellular HMGB1, cells were washed after in vitro treatment with AuNH‐2‐PTT, and a mouse HMGB1 ELISA Kit was used to quantitate the HMGB1 levels. To determine the levels of intracellular ATP, cells were washed after in vitro treatment with AuNH‐2‐PTT, and an ATP Assay Kit (ab83355, Abcam) was used to quantitate ATP levels. Additionally, immunofluorescence was used to visualize calreticulin and HMGB1 expression in the cells. Cells were fixed with 3.7% paraformaldehyde in PBS for 15 min, permeabilized with 0.25% Triton X‐100 in PBS for 15 min at room temperature, and then blocked with 2% BSA in PBS for 1 h. Finally, the cells were incubated with anti‐calreticulin (ab92516, Abcam) and anti‐HMGB1 antibodies (ab79823, Abcam) for 1 h. The cells were then incubated with the corresponding secondary antibody (Alexa Fluor 488, eBioscience) for detection, and the nuclei were stained with DAPI for 15 min.

### Release of Anti‐PD‐L1 Antibodies from AuNH‐2‐Ab In Vitro Measured Using a Transwell System

Hep55.1c cells (5 × 10^4^ in pre‐well) were cultured on microscope slide coverslips in 24‐well plates for 24 h. Cells were co‐cultured with aliquots of FBS‐free medium containing AuNH‐2‐Ab (50 µg mL^−1^) and free anti‐PD‐L1 (10 µg mL^−1^) in the transwell system for 24, 72, and 120 h. After 24 h, the AuNH‐2‐Ab‐treated cells were irradiated with an NIR laser (808 nm, 0.2 W cm^−2^, 10 min). The cells were then stained with DAPI (nucleus) and Vybrant DiD Cell‐Labeling Solution (Invitrogen, Thermo Fisher Scientific) (cell membrane) for visualization. The release of the anti‐PD‐L1 secondary antibodies from AuNH‐2‐Ab was visualized using CLSM (Carl Zeiss LSM 510, Carl Zeiss, Jena, Germany) and flow cytometry (Novocyte Flow Cytometer, ACEA Biosciences).

### Establishment of Syngeneic Orthotopic Mouse Model

A syngeneic hepatocellular carcinoma (HCC) mouse model was established using 8‐week‐old immunocompetent C57BL/6J male mice^[^ ^29^ ^]^ (median weight, 23–28 g) obtained from the National Laboratory Animal Center (Taiwan). The mice underwent midline laparotomy under isoflurane anesthesia, and 2 × 10^6^ Hep55.1c‐Luc cells were directly injected into the left liver lobe using a 30‐gauge needle. The abdominal cavity was closed in two layers. The mice were monitored for overall survival, and the tumor load was recorded upon death. The animals were housed individually in ventilated cages with corncob bedding at a constant temperature of 22 ± 1 °C and a 12 h light‐dark cycle in a specific pathogen‐free environment at China Medical University (Taichung, Taiwan). All animal procedures were conducted in accordance with the relevant guidelines and regulations and were approved by the Institutional Animal Care and Use Committee of the National Yang‐Ming Chiao Tung University (Approval No: 108 055).

### Therapeutic Efficacy and Survival Study After Photoimmunotherapy

The therapeutic effect of various treatments was evaluated in Hep55.1c‐Luc tumor‐bearing mice that were randomly divided into six groups: 1) control (200 µL IgG); 2) anti‐PD‐L1 (100 µg anti‐PD‐L1); 3) AuNH‐2 (15 mg kg^−1^ AuNH‐2); 4) AuNH‐2 (15 mg kg^−1^) plus anti‐PD‐L1 (100 µg); 5) AuNH‐2‐Ab (15 mg kg^−1^); and 6) AuNH‐2‐Ab (15 mg kg^−1^) plus MN groups, and administered the corresponding treatment via intravenous injection into the right femoral vein (q4d × 3) on the seventh days after tumor inoculation.

After 24 h of drug administration, the mice were anesthetized with 4% isoflurane, and the tumor‐bearing region of each mouse was irradiated with an NIR laser (808 nm) at a power density of 0.2 W cm^−2^ for 10 min. Body weights and gross hepatoma pictures were recorded once the mice died. The tumor volume in each treatment group was calculated using the formula: V = 1/2 (L × W^2^), where V represents the tumor volume, L the length, and W the width normalized to body weight for comparison.

To monitor the temperature change during irradiation, the methods previously published by Liu et al. and West et al were adapted. In brief, the interstitial monitoring of PTT was achieved using a K‐thermocouple temperature probe (LP‐35, MISUMI, USA) connected to a temperature logger (TD‐4C, YOTEC, Taiwan).^[^ ^30^ ^]^ To perform the measurement, the thermocouple was first sterilized with 75% ethanol. During NIR irradiation (0.2 W cm^−2^ for 10 min), the thermocouple was inserted into the tumor at 1.5 mm away from the focused laser zone. The readout on the temperature logger was used to plot the temperature–time profile.

For tumor histopathology, formalin‐fixed, and paraffin‐embedded liver tissues sections of 4 µm thickness were prepared and stained with hematoxylin and eosin (H&E). Bioluminescence imaging of the mice was conducted by intraperitoneally injecting the mice under isoflurane anesthesia with d‐luciferin (100 mg kg^−1^) and imaging them using an in vivo imaging system (IVIS 200 System, Xenogen). Kaplan–Meier survival analysis was performed and the median survival times are reported with a 95% confidence interval. The significance of the differences between the six groups was determined by log‐rank analysis.

### Tunel Assay

Cell apoptosis was assayed by immunohistochemistry using a commercial TUNEL Staining Kit (DeadEnd Fluorometric TUNEL System; Promega), as previously described.^[^ ^31^ ^]^ The percentage of TUNEL‐positive cells was expressed as a ratio of the number of TUNEL‐positive cells divided by the total number of nuclei stained with DAPI.

### Preparation of ^125^I‐Radioisotope‐Labeled AuNH‐2‐Ab Nanomedicine

^125^I‐AuNH‐2‐Ab was prepared by mixing ^125^I (1 mL) with AuNH‐2‐Ab, and incubating the mixture on a rotary shaker (300 rpm) at 37 °C for 10–30 min. The labeled AuNH‐2‐Ab was purified by centrifugation at 14 000 rpm for 10 min using the 10 k centrifuge tubes (Sartorius Vivaspin, Germany). Subsequently, the supernatant was removed, and the nanoparticles were resuspended in normal saline and the purification was repeated once. The radiolabeling yield and radiochemical purity were determined using an instant thin‐layer chromatography system (AR‐2000 radio‐TLC Imaging Scanner, USA).

### In Vivo Nano‐PET/CT Scan and Biodistribution of ^125^I‐ AuNH‐2‐Ab

For the investigation of in vivo biodistribution, the distributions of ^125^I‐ AuNH‐2‐Ab nanoparticle (activity equivalent to 37 MBq) in mice were evaluated using nano‐Positron Emission Tomography‐Computed Tomography (nano‐PET/CT). ^125^I‐AuNH‐2‐Ab (in 100 µL of normal saline) was injected through the femoral vein with or without magnetic navigation (MN). PET and CT images were acquired using a NanoScan PET/CT scanner (Mediso, Arlington). PET images were acquired immediately following administration of the ^125^I‐ AuNH‐2‐Ab. Immobilization of the mice was ensured through inhalation of anesthetic isoflurane (3‐4%). Following the acquisition of the PET images, CT images were acquired (X‐ray source: 70 kV, 1 mA; 256 projections) at the same position. These images were fused together and analyzed further using the InVivoScope software. In addition, after 24 h injection, the mice were sacrificed using CO_2_. The major organs were excised, weighed, and assayed for radioactivity using a gamma counter (1470 Wizard; PerkinElmer). Tissue activity was displayed as a percentage of the injected dose per gram of tissue (%ID g^−1^).

### Magnetic Navigation (MN)

The design and experience of the first phase I human clinical trial that used magnetic drug targeting were adopted.^[^ ^32^ ^]^ A cylindrical neodymium magnet (diameter = 8 mm and height = 2 mm) with a magnetic field of 2200 Gauss to the skin nearest to the tumor using bandages, as previously described was fixed.^[^ ^33^ ^]^ MN was applied to mice at 4 h after treatment.

### Evaluation of Anti‐Metastasis Ability

Hep55.1c‐Luc tumor‐inoculated mice were examined for lung metastasis by counting the metastatic nodules in the lungs. After euthanasia, the lungs were excised from the mice, washed once in water, fixed in 4% PFA, and further dehydrated in 30% sucrose at room temperature. Surface metastases appeared as white nodules and were counted under a microscope.

### Isolation of Tumor‐Infiltrating Leukocytes (TILs) and Splenic Cells

Four weeks after tumor inoculation, the tumors and spleen were harvested from the freshly euthanized mice. TILs were isolated and single‐cell suspensions were prepared as previously described.^[^ ^34^ ^]^ In brief, TILs were isolated by digesting the tumor tissue with collagenase type IV (2.5 mg mL^−1^, Gibco) for 20 min and concentrated by centrifugation in a discontinuous Percoll gradient (GE Healthcare). The total number of infiltrating CD8^+^ *T*‐cells per gram of tumor was obtained by multiplying the percentage of CD8^+^ *T*‐cells by the total number of lymphocytes obtained from the Percoll gradient and dividing that number by 100 and by the weight of the tumors. TAMs (CD11b^+^CD206^+^F4/80^+^ cells) in TIL suspensions were analyzed using a FACS Aria (BD Biosciences) after staining with anti‐CD11b, anti‐CD206, or anti‐F4/80 antibodies (purity >95%). Tregs (CD4^+^CD25^+^Foxp3^+^) were isolated using a Treg Isolation Kit (Miltenyi Biotec) (purity >90%). In some experiments, the CD4^+^CD25^+^ *T*‐cells were purified using a FACS Aria (BD Biosciences) after staining with anti‐CD4 and anti‐CD25 antibodies (purity >95%). The spleens were teased and filtered through a nylon mesh to obtain single‐cell suspensions. Red blood cells were removed by treating the cell suspension with red blood cell lysis buffer to obtain pure splenocyte single‐cell suspension.

### Flow Cytometry

Cells were stained with fluorochrome‐conjugated monoclonal antibodies corresponding to the cell surface markers as follows: anti‐PD‐L1 (MIH5), anti‐CD3 (145‐2C11), anti‐CD45 (30‐F11), anti‐CD8 (53‐6.7), anti‐CD11c (M1/70), anti‐CD40 (3/23), anti‐CD80 (16‐10A1), anti‐CD86 (GL1), anti‐CD11b (M1/70), anti‐NK1.1 (PK136), anti‐CD45 (30‐F11), anti‐IFN‐γ (XMG1.2), anti‐CD44 (IM7.8.1R), anti‐CD4 (GK1.5), anti‐CD25 (PC61.5), anti‐F4/80 (BM8), anti‐CD206 (MR5D3), and 7‐AAD (all purchased from BD). As control, cells were stained with mouse IgG1 isotype control antibodies. Flow cytometric acquisition was performed using a FACScan (BD Biosciences) and the data were analyzed using CellQuest (BD Biosciences) and FlowJo (ver. 8.8, Tree Star Inc.) softwares.

The gating strategies were based on the justification of the first gate, exclusion of doublets by FSC‐A and FSC‐H, and exclusion of dead cells by selection of 7‐AAD^−^/CD45^+^ as described previously.^[^ ^35^ ^]^ Then, CD8^+^ *T*‐cells, CD4^+^ T cells, Tregs, TAMs in TILs, tumor‐infiltrated dendritic cells (DCs), tumor‐infiltrated natural killer cells (NKs), or splenocyte suspensions were analyzed using multicolor flow cytometry (FACScan, BD) with CellQuest (BD Biosciences) and FlowJo (ver. 8.8, Tree Star Inc.) softwares. The results are presented as the percentage of positively stained cells relative to the total number of cells.

### Evaluation of Granzyme B Expression in CD8^+^ *T*‐Cells

For intracellular staining of granzyme B, TILs were cultured in the presence of anti‐CD3 (1 µg mL^−1^) for 48 h. The cells were then incubated with an anti‐CD8 prior to permeabilization with Triton X‐100 and then stained with an anti‐granzyme B (Millipore).

### Immunocytochemical Analysis

Hep55.1c‐Luc cells seeded on chamber slides were fixed for 15 min at room temperature with 4% paraformaldehyde. The cells were incubated with primary antibodies (Phalloidin, anti‐PD‐L1 Invitrogen) at 4 °C overnight. Fluorophore‐conjugated secondary antibodies (Alexa Fluor 488‐labeled anti‐rabbit secondary antibodies, Invitrogen) and DAPI (Sigma–Aldrich) were treated for 1 h at 25 °C. A CLSM (Carl Zeiss LSM 510, Carl Zeiss, Jena, Germany) was used to obtain images of the cells (performed in a blinded manner to the treatment groups). ImageJ software was used to analyze the fluorescence intensity by examining the mean intensity of each selected area (a minimum of ten rectangular areas were analyzed).

### Immunohistochemical Analysis

Animals were anesthetized with chloral hydrate (0.4 g kg^−1^, ip), and their abdominal skin tissues were fixed by transcardial perfusion with saline, followed by immersion in 4% paraformaldehyde. The tissue samples were dehydrated in 30% sucrose, and frozen on dry ice, and a series of adjacent 6 µm‐thick coronal sections were cut using a cryostat. The sections were stained with H&E and observed under a light microscope (Nikon E600). Each section was immunostained by incubating with primary antibodies against CD3 (1:100; BD), CD4 (1:400; BD), and CD8 (1:400; BD) and corresponding secondary antibodies conjugated with FITC or Cy‐3 (1:500; Jackson Immunoresearch). The sections were analyzed using a CLSM (Carl Zeiss LSM 510, Carl Zeiss, Jena, Germany). For IgG deposition in the glomeruli, kidney sections were stained with a goat anti‐mouse IgG antibody (Invitrogen).

### Assessment of irAEs

A irAEs were evaluated by analyzing 1) changes in body weight, 2) histological assessment (H&E staining), and 3) liver and kidney function after treatment with the nanomedicine. The body weights of the mice were monitored during the treatment. In addition, tissue sections of the liver, heart, lungs, spleen, kidneys, and colon of the mice treated with each nanomedicine were stained and histologically evaluated four weeks after tumor inoculation (*n* = 6). Furthermore, the biochemical profile of ALT, AST, creatinine, and glucose in the mouse serum was measured at sequential time points (1, 7, 14, 21, and 28 days) in each treatment group (*n* = 6) using a Beckman Unicell DxC800 analyzer.

### Statistical Analysis

Statistical analysis was performed using Prism 9.0. All in vitro experiments were repeated at least three times (*n* = 3, biologically independent experiments). Data are expressed as mean ± standard deviation (SD) unless otherwise noted. Comparisons between two groups were performed using an unpaired two‐tailed *t*‐test. When comparing multiple groups, one‐way analysis of variance (ANOVA) followed by appropriate *post‐hoc* test (Tukey's or Brown‐Forsythe *post‐hoc* tests) were performed. Kaplan‐Meier survival curves were analyzed using the log‐rank test. Statistical significance was set at *p*‐value < 0.05.

## Conflict of Interest

The authors declare no conflict of interest.

## Supporting information

Supporting Information

## Data Availability

The data that support the findings of this study are available from the corresponding author upon reasonable request.
