# Supplementary material for: Manipulating the Crosstalk between Cancer and Immunosuppressive Cells with Phototherapeutic Gold‐Nanohut for Reprogramming Tumor Microenvironment
Source: Adv Sci (Weinh). 2024 Jun 23;11(32):2404347. doi: 10.1002/advs.202404347 (PMC11348132; doi:10.1002/advs.202404347)
Supplement: Supplementary file 1 — Supporting Information [file ADVS-11-2404347-s001.docx]

**Manipulating the Crosstalk between Cancer and Immunosuppressive Cells with Phototherapeutic Gold-Nanohut for Reprogramming Tumor Microenvironment**

Hung-Wei Cheng^1^, Wei Lee^2^, Fei-Ting Hsu^3^, Yen-Ho Lai^2^, Shu-Rou Huang^4^, Chris Seh Hong Lim^5^, Zhen-Kai Lin^1^, Shih-Chao Hsu^6^, Chih-Sheng Chiang^2,7,8^ *, Long-Bin Jeng^2,9,10^ *, Woei-Cherng Shyu^4,7,8^ * and San-Yuan Chen^1,7,11^ *

^1^ Department of Materials Science and Engineering, National Yang Ming Chiao Tung University, Hsinchu, Taiwan

e-mail: sanyuanchen@nycu.edu.tw

^2^ Cell Therapy Center, China Medical University Hospital, Taiwan

e-mail: brian.chiang@mail.cmu.edu.tw

^3^ Department of Biological Science and Technology, China Medical University, Taichung, Taiwan

e-mail: sakiro920@mail.cmu.edu.tw

^4^ Translational Medicine Research Center, Drug development Center and Department of Neurology, China Medical University & Hospital, Taichung, Taiwan

e-mail: shyu9423@gmail.com

^5^ Department of Physician Assistant Studies, School of Health and Rehabilitation Sciences, MGH Institute, Massachusetts, USA

e-mail: chrislim@mghihp.edu

^6^ Department of Surgery, China Medical University Hospital, Taichung, Taiwan

e-mail: 012722@tool.caaumed.org.tw

^7^ Graduate Institute of Biomedical Science, China Medical University, Taichung, Taiwan
e-mail: brian.chiang@mail.cmu.edu.tw

^8^ Neuroscience and Brain Disease Center, China Medical University, Taichung, Taiwan

e-mail: brian.chiang@mail.cmu.edu.tw

^9^ Organ Transplantation Center, China Medical University Hospital, Taichung, Taiwan

e-mail: longbin.cmuh@gmail.com

^10^ School of Medicine, China Medical University, Taichung, Taiwan

e-mail: longbin.cmuh@gmail.com

^11^ School of Dentistry, College of Dental Medicine, Kaohsiung Medical University, Taiwan

e-mail: sanyuanchen@nycu.edu.tw

* Corresponding author: Chih-Sheng Chiang, Long-Bin Jeng, Woei-Cherng Shyu and San-Yuan Chen contributed equally

**
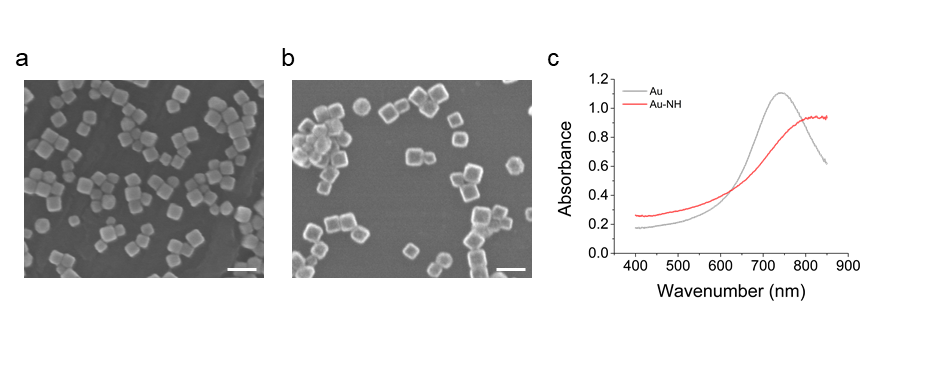
**

**Figure S1.** The SEM image of (a) silver nanocages (AgNCs) and (b) gold nanocages (AuNCs). Scale bar= 100 nm

**

**

**Figure S2.** The Zeta potential of AuNH@F (fucoidan-coated AuNH) and AuNH@F/IO (AuNH-1).

**
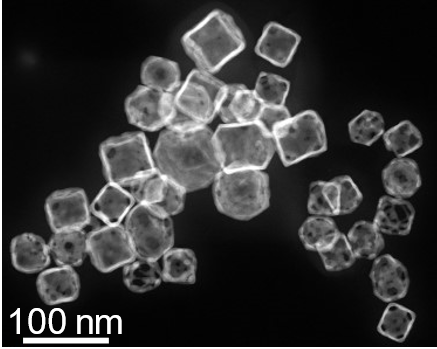
**

**Figure S3.** Representative TEM images of AuNH-2-Ab demonstrated homogeneous size and structure under transmission electron microscopy (TEM) at low magnification of 300 kX.

**
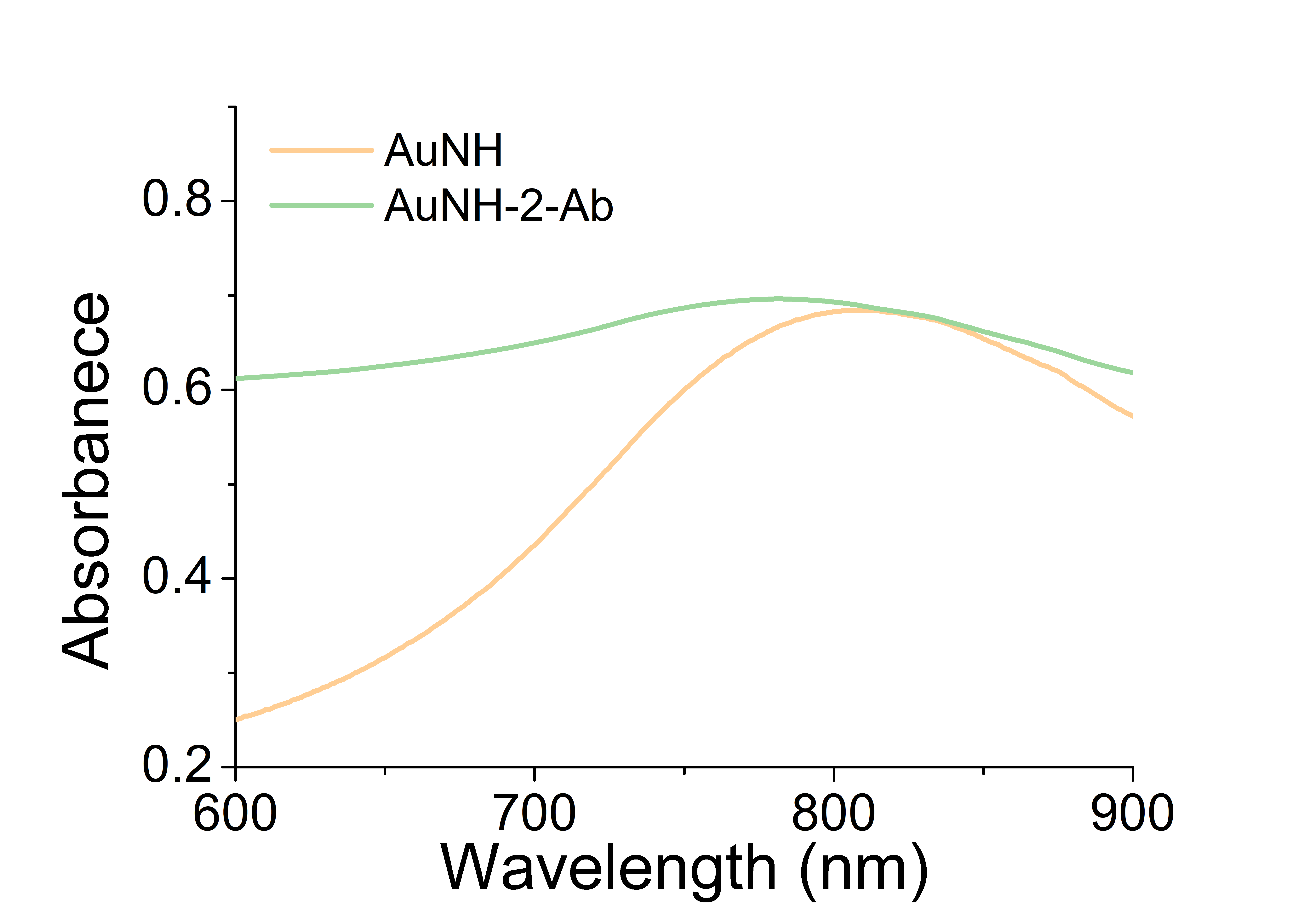
**

**Figure S4.** UV- visible spectrum of AuNH and AuNH-2-Ab.

**
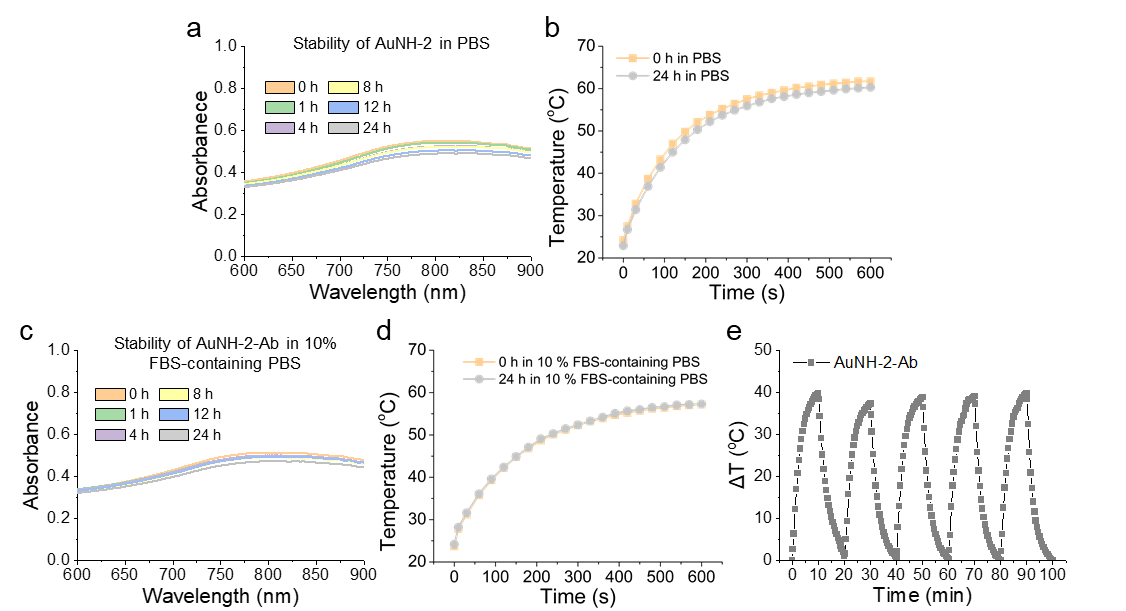
**

**Figure S5.** (a) Localized surface plasmon resonance (LSPR) spectra of AuNH-2 at 0, 1, 4, 8, 12, and 24 h of incubation in PBS. (b) The temperature-time profile of AuNH-2 in PBS at 0 and 24 h under NIR (808 nm) irradiation for 10 min at a power density of 0.5 W cm^-2^. (c) LSPR spectra of AuNH-2-Ab at 0, 1, 4, 8, 12, and 24 h of incubation in 10% FBS-containing PBS. (d) The temperature-time profile of AuNH-2 in 10% FBS-containing PBS at pH 7.4 at 0 and 24 h under NIR (808 nm) irradiation for 10 min at a power density of 0.5 W cm^-2^. (e) AuNH-2-Ab was dispersed in 10% FBS-containing PBS and irradiated with NIR for 5 cycles. In each cycle, the AuNH-2-Ab was exposed to NIR at 0.5 W cm^-2^ laser for 10 min and allowed for 10 min break at room temperature.

**
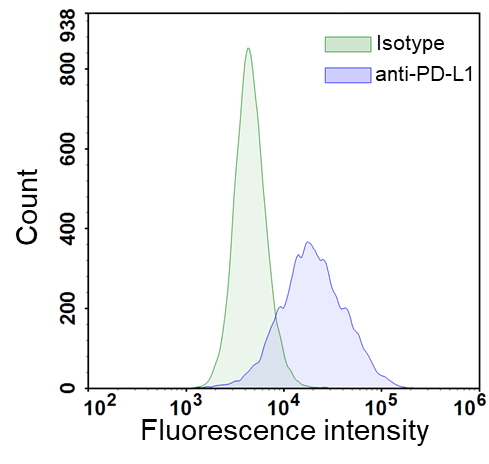
**

**Figure S6.** Hep55.1c expresses PD-L1 on the surface.


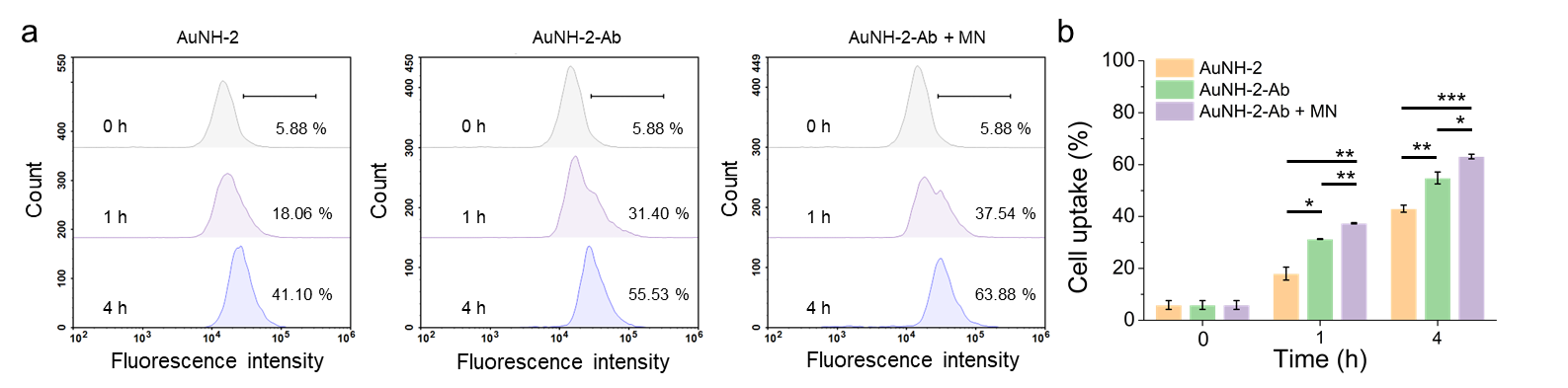


**Figure S7.** Quantitative results of the percentage of cell uptake when the cells were incubated with AuNH-2, AuNH-2-Ab and AuNH-2-Ab +MN for 0, 1 and 4 h using flow cytometry.


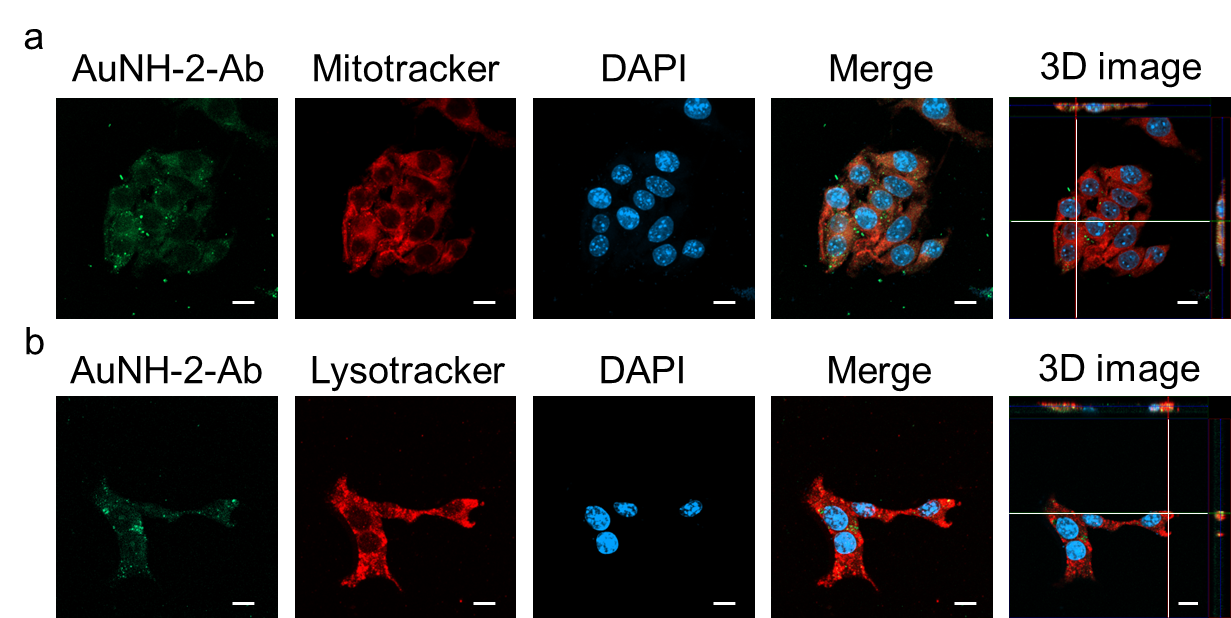


**Figure S8.** Cell trafficking of AuNH-2-Ab using (a) Mitotracker and (b) lysotracker at 12 h incubation. Scale bar = 10 μm.

**
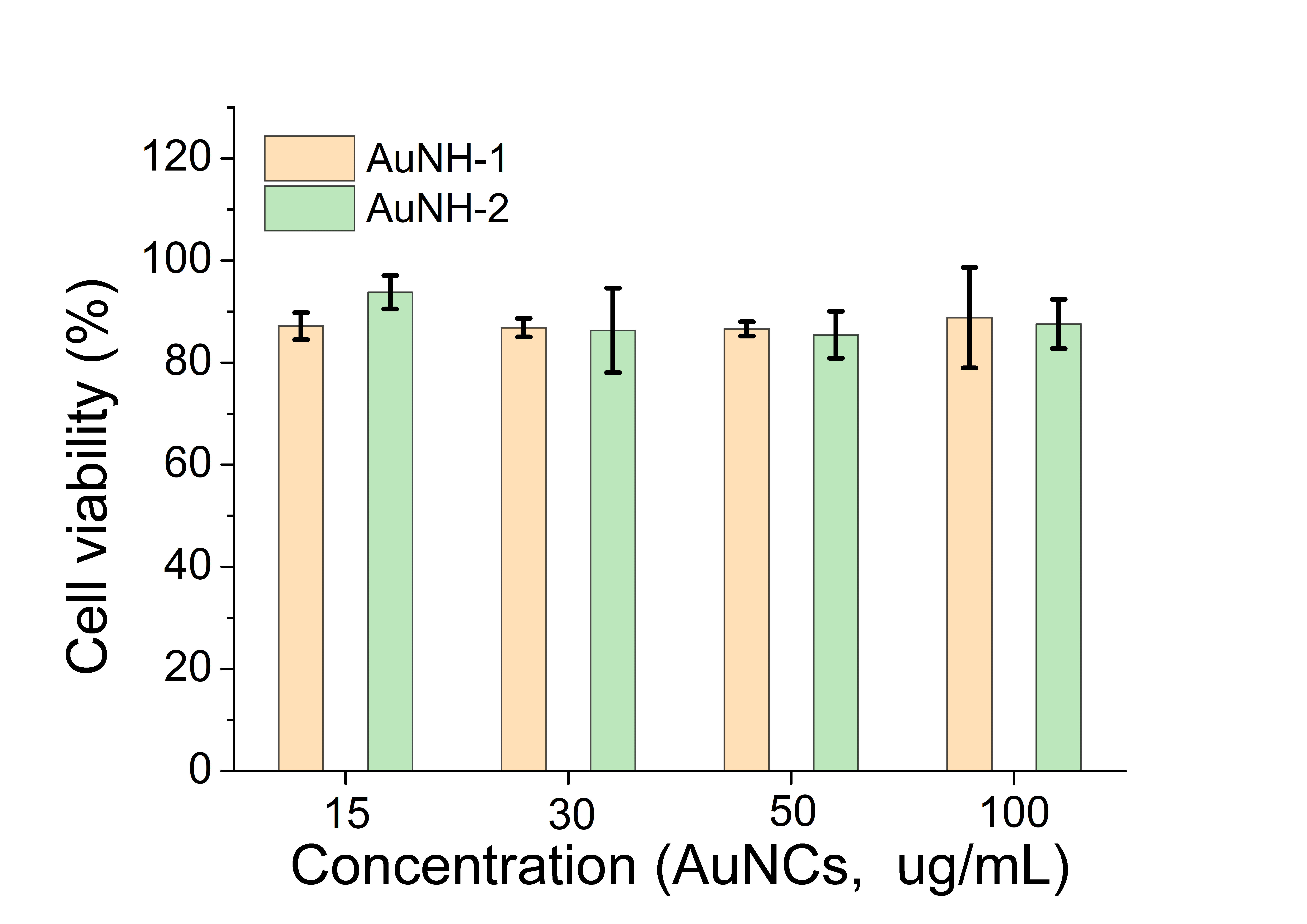
**

**Figure S9.** Cytotoxicity of AuNH-1 and AuNH-2 at different concentrations (15, 30, 50, 100 μg mL^-1^). n = 3 biologically independent samples.

**
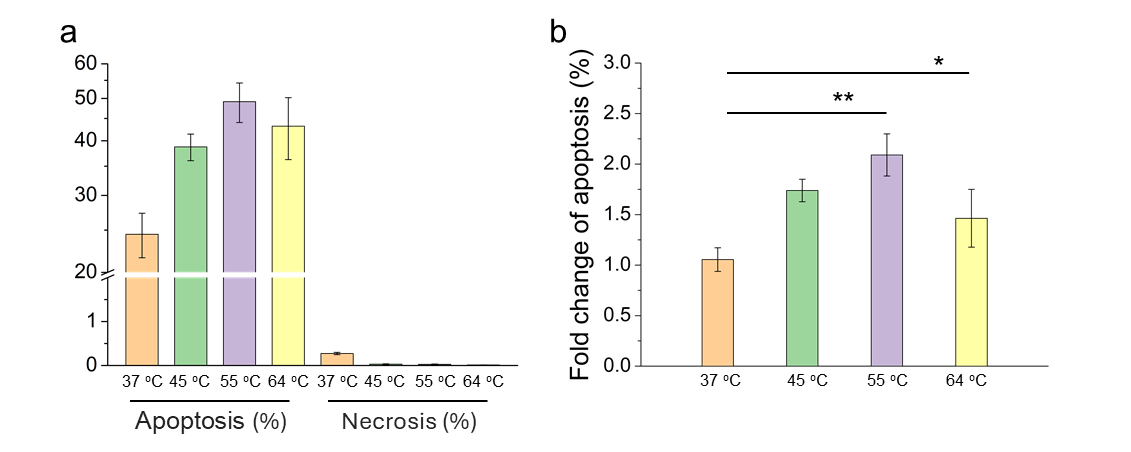
**

**Figure S10**. (a) Cell apoptosis and necrosis, (b) Fold change of apoptosis in the Hep 55.1c cells treated with 37 ^o^C (control), 45 ^o^C (50 μg mL^-1^ AuNH-2, 0.2 W cm^-2^), 55 ^o^C (50 μg mL^-1^ AuNH-2 ,0.5 W cm^-2^) and 64 ^o^C (100 μg mL^-1^ AuNH-2 ,0.5 W cm^-2^) (n = 3). One-way ANOVA with the Bonferroni post hoc test. *P < 0.05, **P < 0.01.

**
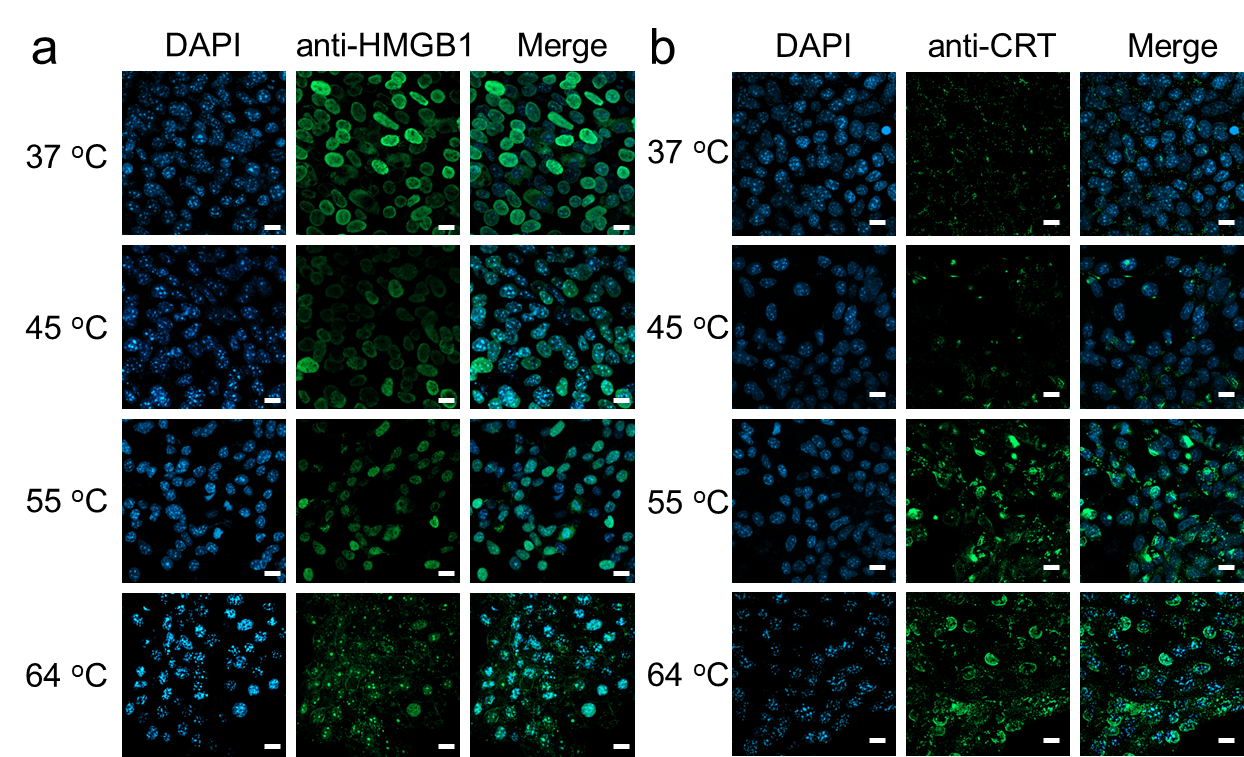
**

**Figure S11.** The immunofluorescent images of the ICD markers including (a) high-mobility group box 1 (HMGB1) and (b) calcium reticulin (CRT) after the Hep55.1c hepatocellular carcinoma cells were treated with the photo-hyperthermia of AuNH-2 at 45, 55 and 64 ^o^C. (Scale bar= 10 μm)

**
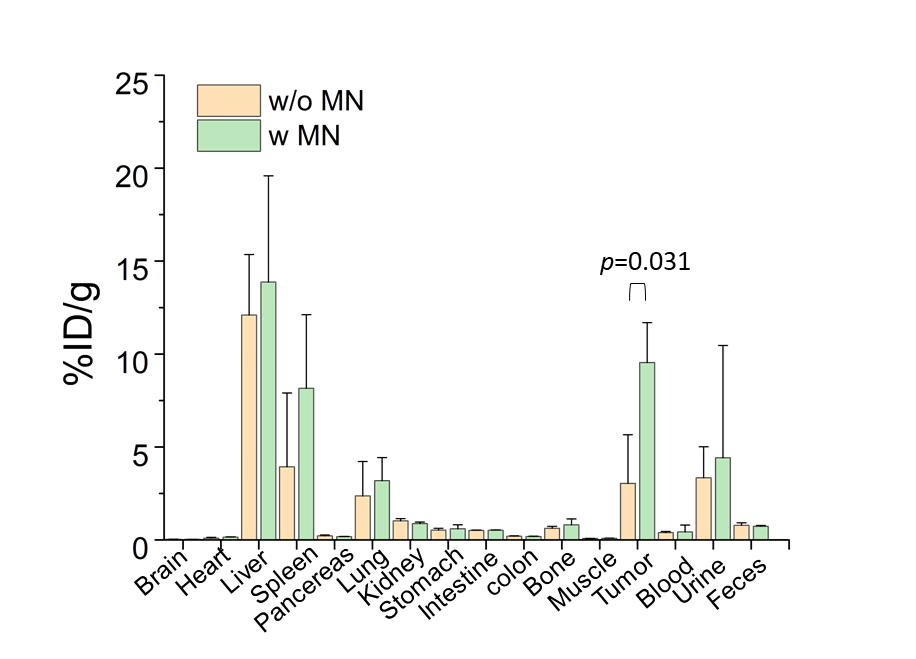
**

**Figure S12.** Quantitative analysis of uptake intensity in each distributed major organ post-injection of ^125^I-AuNH-2-Ab with or without magnetic navigation at 24 h post treatment.

**
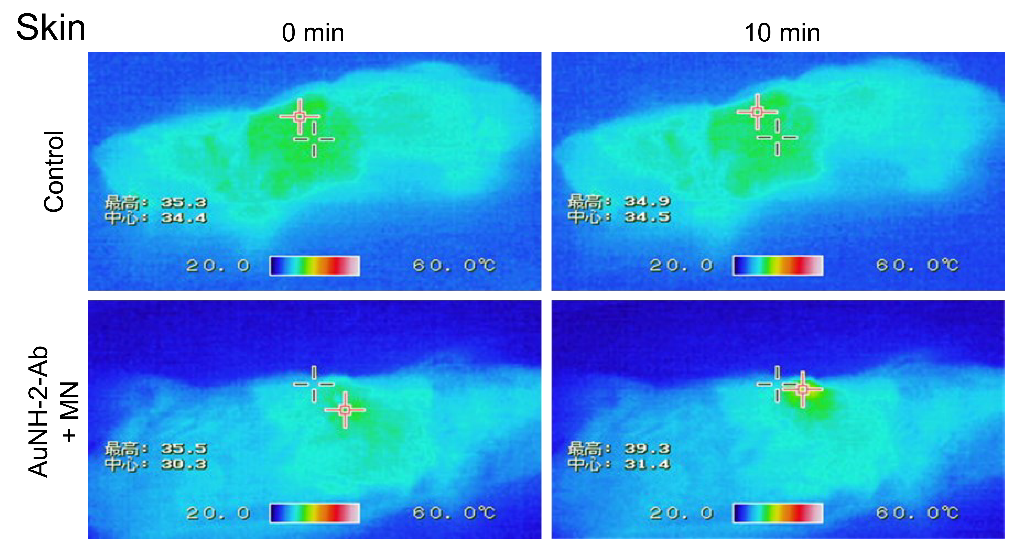
**

**Figure S13.** Analysis of observed temperature in representative thermo-image of skin exposed in NIR irradiation in control (PBS) and AuNH-2-Ab plus MN (0.2 W cm^-2^).

**
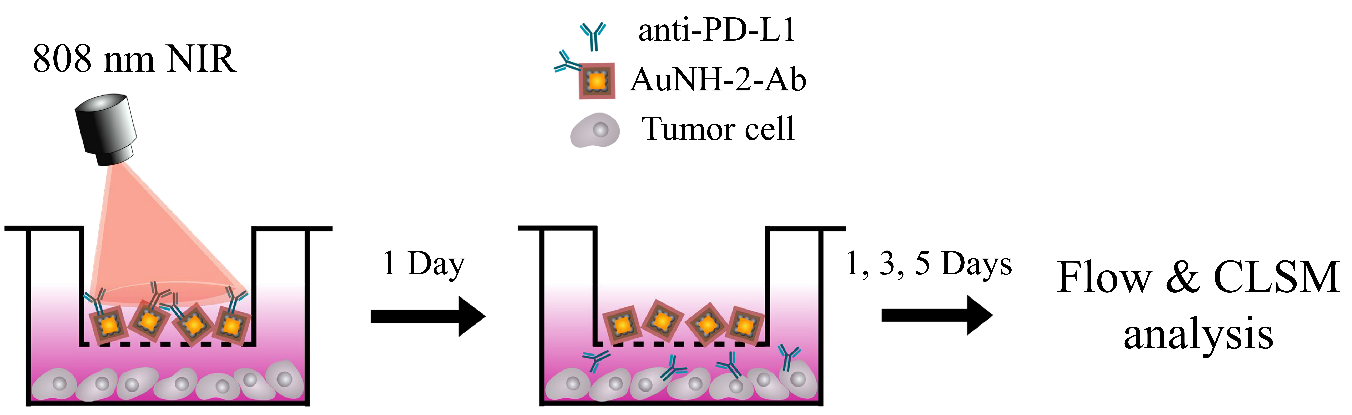
**

**Figure S14.** Scheme showing the AuNH-2-Ab co-cultured with tumor in the transwell system experiment. AuNH-2-Ab and free anti-PD-L1-PE were placed in the upper chamber, and high-level PD-L1 tumor cells were cultured in the lower chamber.

**
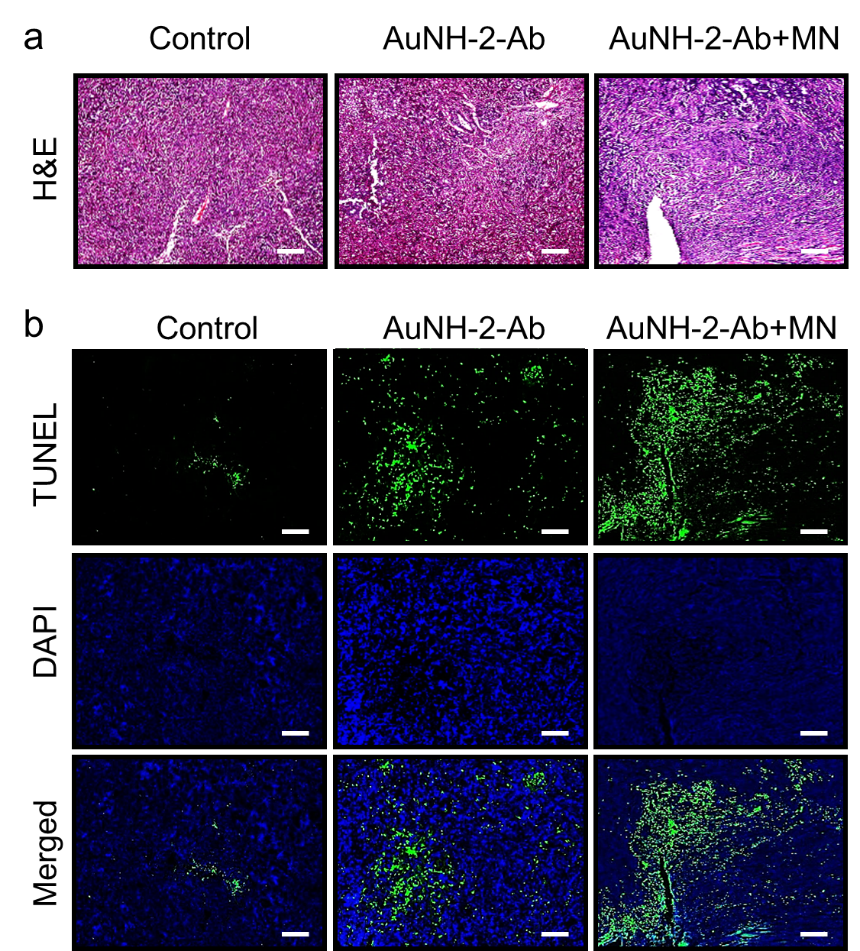
**

**Figure S15.** (a) Hematoxylin and eosin (H&E) stain and (b) TUNEL staining for the tumors after the mice were treated with control (IgG), AuNH-2-Ab, and AuNH-2-Ab + MN (Scale bar= 50 μm).

**
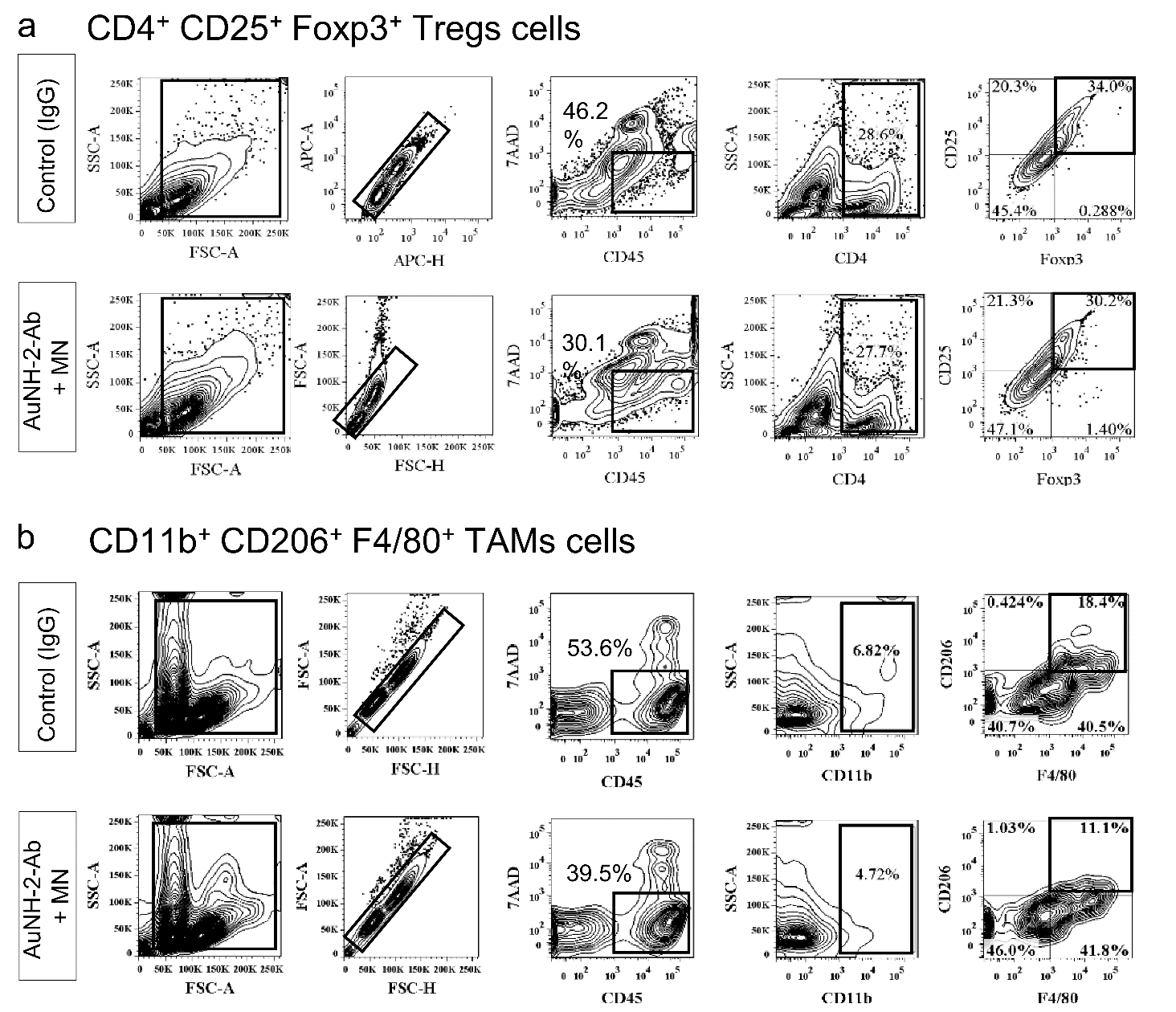
**

**Figure S16.** Analysis of gating strategy to determine the Tregs and TAMs in control (IgG) and AuNH-2-Ab plus MN.


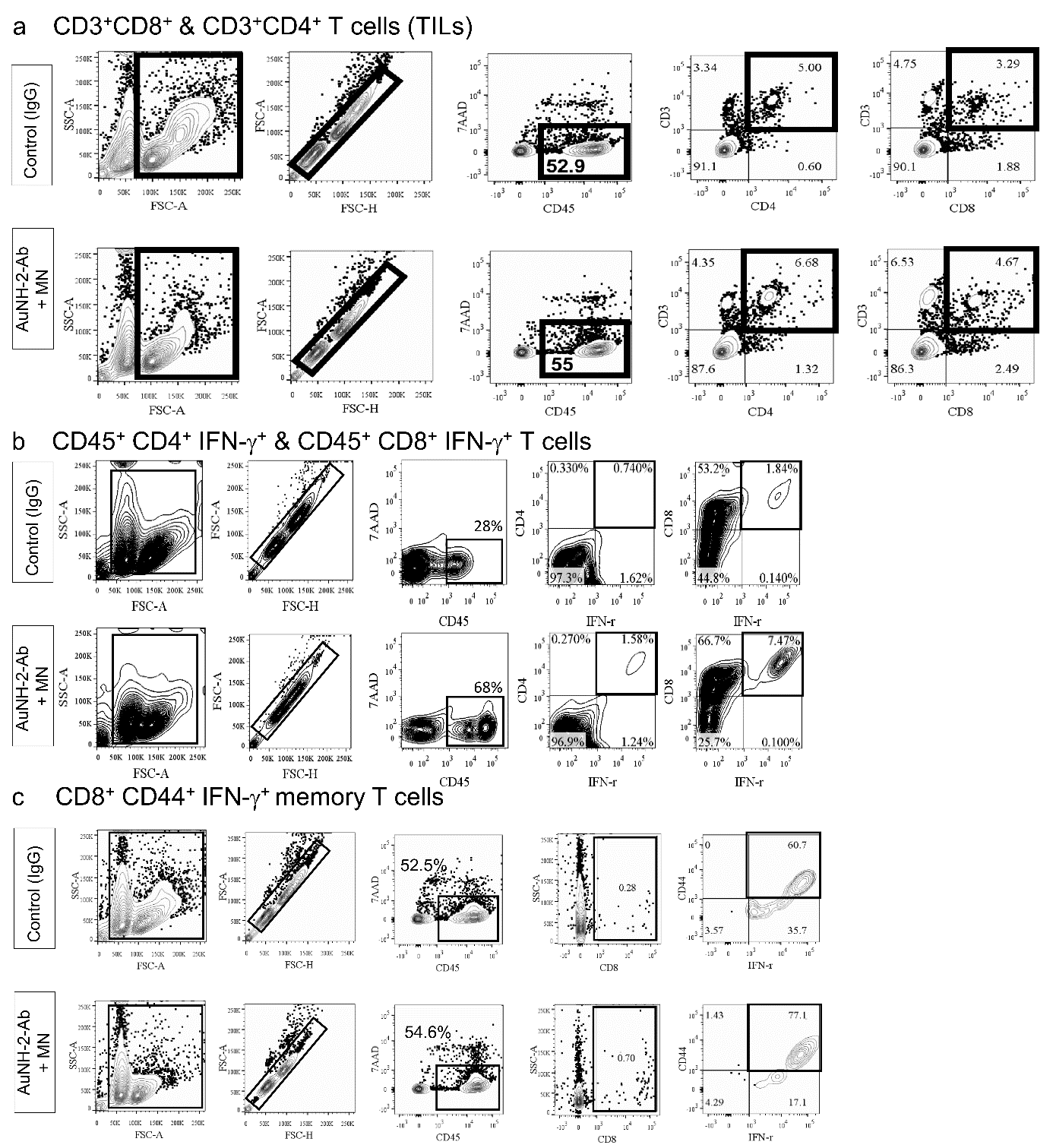


**Figure S17.** Analysis of gating strategy to determine the tumor infiltrative leukocytes (TILs) and memory T cell in control (IgG) and AuNH-2-Ab plus MN.


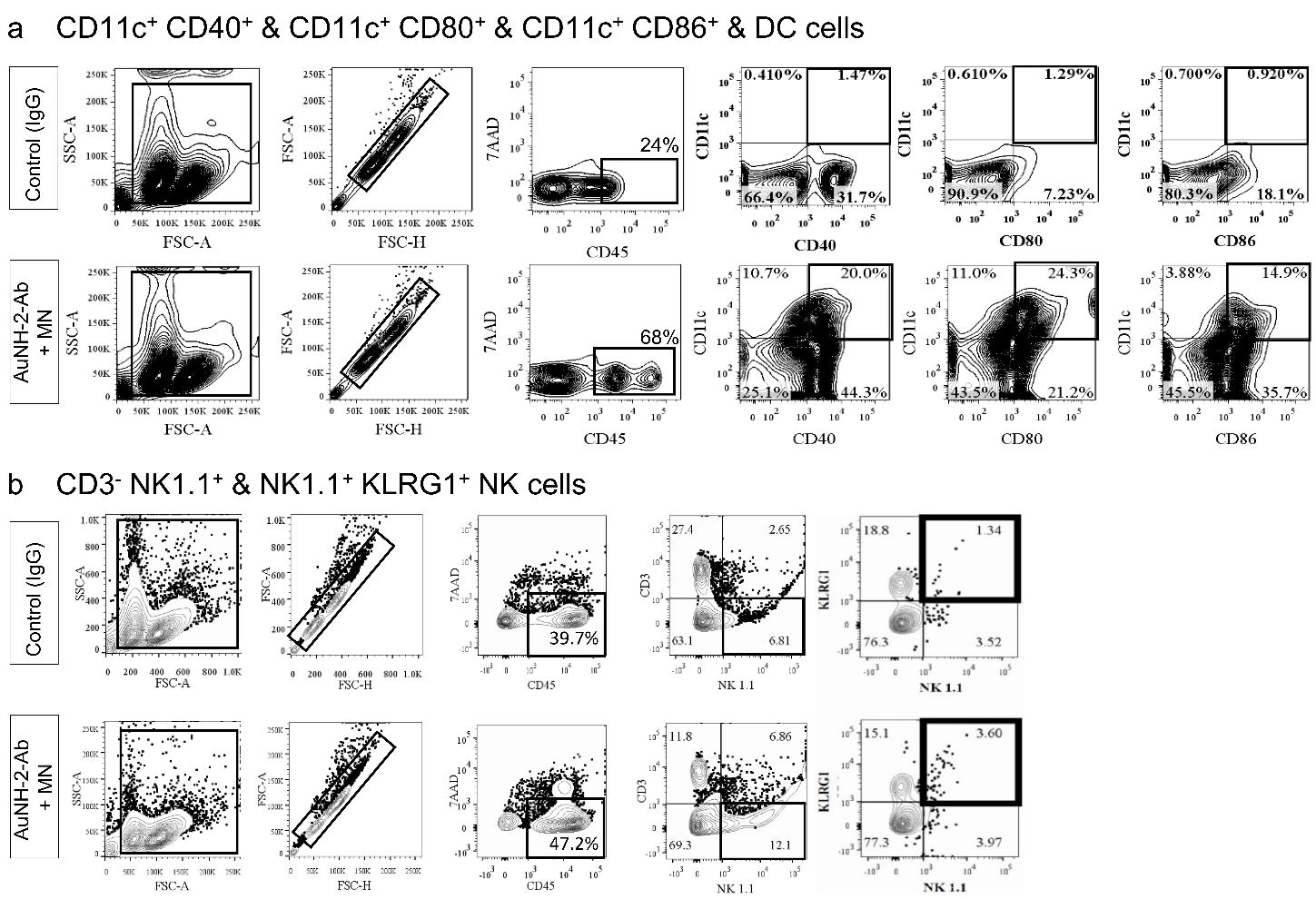


**Figure S18.** Analysis of gating strategy to determine the DC cells and NK cells in control (IgG) and AuNH-2-Ab plus MN.


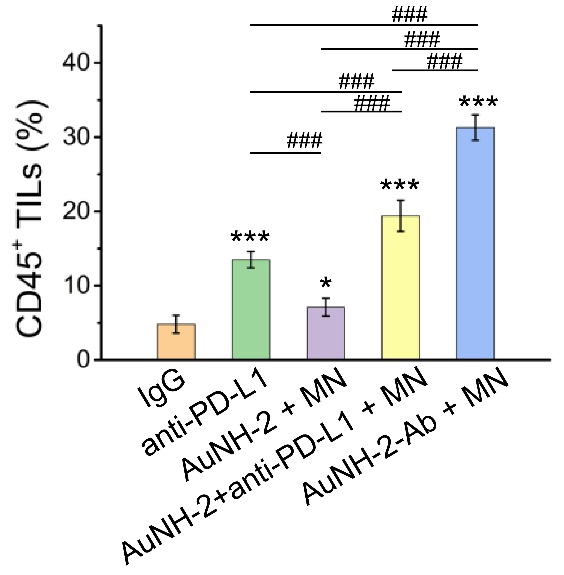


**Figure S19.** Number of CD45^+^ TILs at 4 weeks after tumor inoculation. Quantitative measurement of CD45^+^ TILs at 4 weeks after tumor inoculation treated with IgG, anti-PD-L1, AuNH-2 plus MN, AuNH-2 + antiPDL1 plus MN, and AuNH-2-Ab plus MN. One-way ANOVA with the Tukey’s *post hoc* test; n = 6. *p < 0.05, **p < 0.01, and ***p < 0.001. ^#^p < 0.05, ^##^p < 0.01 and ^###^p < 0.001 between groups.

**
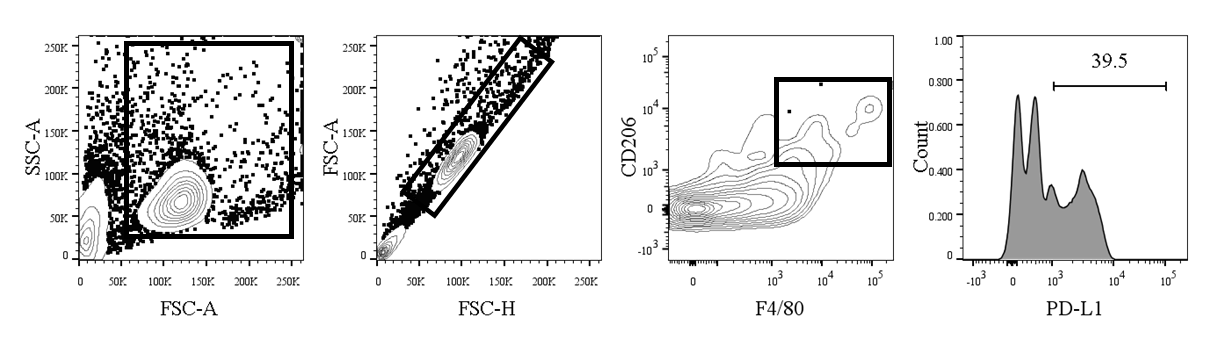
**

**Figure S20.** Analysis of gating strategy to determine the PD-L1 expression of tumor-associated macrophages (TAMs) in tumor immune microenvironment.


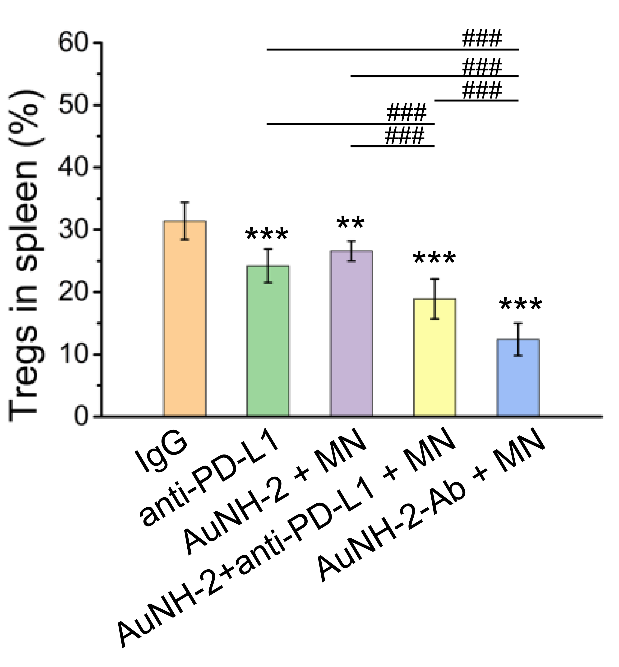


**Figure S21.** Quantitative analysis of immunological profiles of Tregs in spleen in each treated group with or without MN. One-way ANOVA with the Tukey’s *post hoc* test; n = 6. *p < 0.05, **p < 0.01, and ***p < 0.001. ^#^p < 0.05, ^##^p < 0.01 and ^###^p < 0.001 between groups.


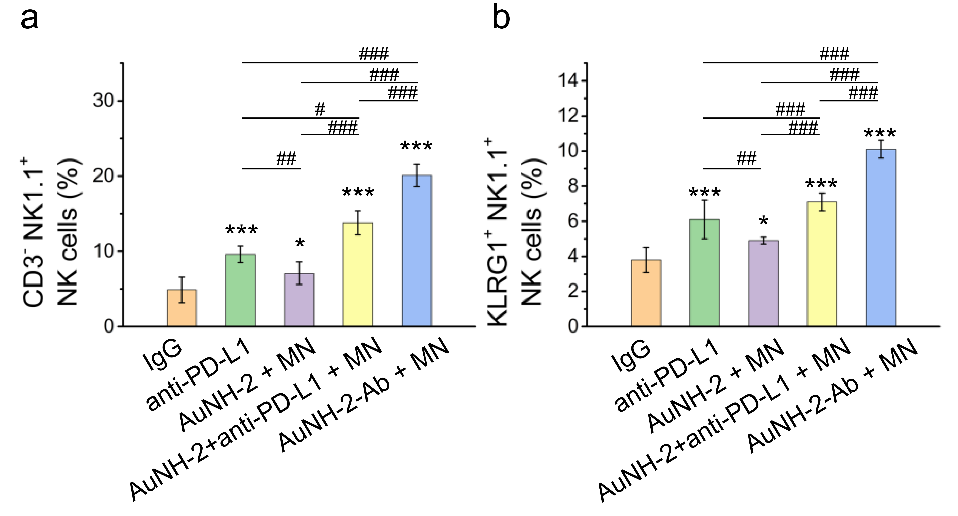


**Figure S22.** Quantitative analysis of antitumor immune cells in TME treated with various formulation including (a) CD3^-^NK1.1^+^ and (b) KLRG1^+^NK1.1^+^ at 4 week after tumor inoculation. One-way ANOVA with the Tukey’s *post hoc* test; n = 6. *p < 0.05, **p < 0.01, and ***p ≤0.001. ^#^p < 0.05, ^##^p < 0.01 and ^###^p < 0.001 between groups.


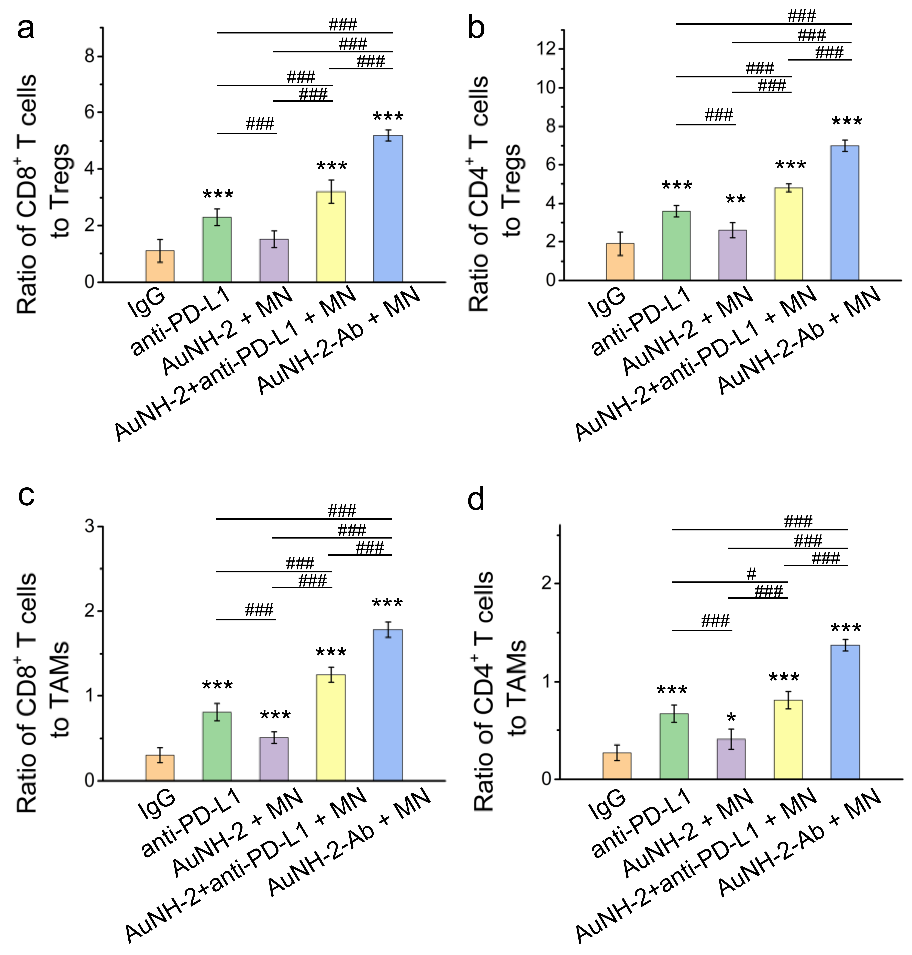


**Figure S23.** Quantitative examination of immune cells in TME at 4 weeks after tumor inoculation including (a) ratio of CD8^+^ T cell to Tregs, (b) ratio of CD4^+^ T cell to Tregs, (c) ratio of CD8^+^ T cells to TAMs and (d) ratio of CD4^+^ T cells to TAMs, after treatment with each combination therapy. One-way ANOVA with the Tukey’s *post hoc* test; n = 6. *p < 0.05, **p < 0.01, and ***p < 0.001. ^#^p < 0.05, ^##^p < 0.01 and ^###^p < 0.001 between groups.


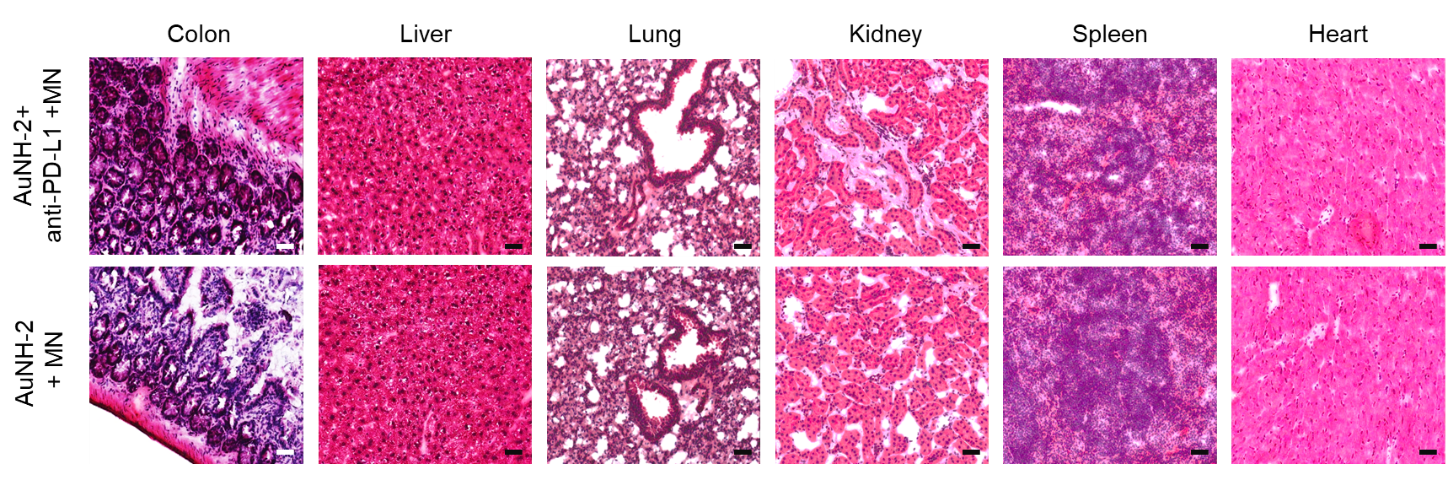


**Figure S24.** Safety assessment using hematoxylin and eosin stain (H&E stain). Histological assessment of major organs using hematoxylin and eosin stain (H&E stain) after treatment with AuNH-2 plus MN, AuNH-2 plus anti-PD-L1 and MN. (Scale bar= 50 μm).
